# Supplementary material for: Attitude of health professionals towards COVID-19 vaccination and associated factors among health professionals, Western Ethiopia: A cross-sectional survey
Source: PLoS One. 2022 Mar 9;17(3):e0265061. doi: 10.1371/journal.pone.0265061 (PMC8906598; doi:10.1371/journal.pone.0265061)
Supplement: S3 File — (DOCX) [file pone.0265061.s003.docx]

**Wollega University**

**Institute of health sciences**

**Department of Public Health**

**Questionnaires (English version)**

**Consent form**

This questionnaire is prepared to collect data on research focusing **“**To assess **Attitude and Perception of health professionals towards COVID-19 vaccine in Nekemte town, Western Ethiopia”.** You have been included randomly to this study and the information you will provide, will neither be disclosed to the third party, nor be used for another purpose and will be rather kept confidential. The information collected will not be linked to you or your family members and you don’t need to tell your/family member/ name. You have full right to participate, reject or not to answer all or part of the questions. However, your participation in giving the right information is quite important for our study.

Do you agree to participate?

Yes

No

Name of data collector____________________ Signature

**For any information contact us on**

**Tadesse Tolossa- +251911936804**

**Gebeyehu Abera- +251917030226**

**Name of health facility __________________________________**

# Part I: Socio-demographic variables

| **Code** | **Characteristics** | **Response** |
| --- | --- | --- |
| 101 | Age | ____ years old |
| 102 | Gender | _1_ Male _2_ Female |
| 103 | Marital status | _1_ Never married _2_ Married  _3_ separated/divorced _4_ Widowed |
| 104 | Ethnicity | _1_ Oromo _2_ Amhara  _3_ Tigray _4_ Other(specify)______ |
| 105 | Religion | _1_ Muslim _2_ Protestant  _3_ Catholic _4_ Orthodox  _5_ Other(specify)______ |
| 105 | Institution | _1_ Hospital _2_ Health center  _3_ Private |
| 106 | Educational level | _1_ Diploma _2_ 1^st^ degree  _3_ Master degree _4_ PhD degree  _5_ Specialty degree |
| 107 | Background/specialty | _1_ Nurse _2_ Midwife _3_ Pharmacist/Druggist  _4_ Medicine _5_ Public Health _6_ Anesthesia  _7_ MLS _8_Psychiatry 9. Env’tal health  10. other (specify) ____________ |
| 108 | Previous vaccination allergy in your life? | _1_ Yes _2_ No |
| 109 | Do you have a chronic illness? | _1_ Yes _2_ No |
| 110 | If your answer to Q109 is yes, select any preexisting medical conditions you have been diagnosed with? | _1_ HTN _2_ Cancer  _3_ HIV/AIDS _4_ Asthma  _5_ Other(**specify**)______________ |

# Part II Knowledge of participants towards COVID-19 vaccine

| **Code** | **Characteristics** | **Response** |
| --- | --- | --- |
| 201 | Do you know about the COVID-19 vaccine? | _1_ Yes _2_ No _3_ I don’t know |
| 202 | Do you know about the effectiveness of COVID-19 vaccine? | _1_ Yes _2_ No _3_ I don’t know |
| 203 | Does vaccination increase allergic reactions? | _1_ Yes _2_ No _3_ I don’t know |
| 204 | Does vaccination increase autoimmune diseases? | _1_ Yes _2_ No _3_ I don’t know |
| 205 | Do developed vaccine protect against variant strains? | _1_ Yes _2_ No _3_ I don’t know |
| 206 | Will COVID-19 vaccine provide long term protection? | _1_ Yes _2_ No _3_ I don’t know |
| 207 | What types of COVID-19 vaccine is available? | _1_ AstraZeneca _2_ Pfizer _3_ Moderna  _4_ Johnson and Johnson _5_ Other(specify)____ |
| 208 | Are you vaccinated with COVID-19 vaccine? | _1_ Yes _2_ No |
| 209 | If no, the reason not to vaccinated? | _1_ Not available _2_ fear of adverse effect  _3_ Unreliable, due to short time for development  _4_ Don’t have enough information  _5_ I prefer other ways of protection  _6_ Vaccine causing COVID-19 _7_ Ineffective  _8_ COVID-19 is not a serious illness  _9_ I don’t like needles _10_ Others ______ |
| 210 | Who should have been vaccinated, do you think? | _1_ Those who have not yet been infected  _2_ People infected with COVID-19  _3_ Newly recovered from COVID-19  _4_ Everyone |
| 211 | Who's supposed to be vaccinated first, you think? | _1_ Elders _2_ Health workers _3_ Students  _4_ Public/ private employee  5. People with chronic disease  6. pregnant women  _7_ Others(specify)_ |
| 212 | Your recommendation about COVID-19 vaccine | _1_ Be optional for all  _2_ Be mandatory for all  _3_ Accessible and available at no cost  _4_ Accessible and available at fair cost  5 Priority should be given for health professional & risk  groups  _6_ No recommendation |
| 213 | Are you willing to accept COVID-19 vaccine? | _1_ Definitely Yes _2_ Probably Yes  _3_ Probably No _1_ Definitely No |

**Part III: Question related to attitude towards COVID-19 vaccine**

**(1= strongly disagree, 2= disagree, 3= neutral, 4= agree, 5= strongly agree)**

| **Code** | **Variables** | **1** | **2** | **3** | **4** | **5** |
| --- | --- | --- | --- | --- | --- | --- |
| 301 | Newly discovered COVID-19 vaccine that is given for Ethiopia is the actual one that the discovered country is using yet |  |  |  |  |  |
| 302 | If one individual is vaccinated it will have great contribution for the other person (society) |  |  |  |  |  |
| 303 | The newly discovered COVID-19 vaccine is safe. |  |  |  |  |  |
| 304 | I will take the COVID-19 vaccine without any hesitation |  |  |  |  |  |
| 305 | I will encourage my family/friends/relatives to get vaccinated |  |  |  |  |  |
| 306 | It is not possible to reduce the incidence of COVID-19 without vaccination. |  |  |  |  |  |
| 307 | The COVID-19 vaccine should be distributed fairly to all of us. |  |  |  |  |  |
| 308 | “Even though I am a religious man I have probability to be infected and for that I have to take the vaccine” |  |  |  |  |  |
| 309 | The way to overcome the COVID‐19  Pandemic is mass vaccination |  |  |  |  |  |
| 310 | The best preventive measure for COVID‐19 is getting Vaccinated |  |  |  |  |  |
| 311 | I think that the vaccine was not tested for enough time |  |  |  |  |  |
| 312 | We can stop precaution after being vaccinated |  |  |  |  |  |
| 313 | Do you have a willing to take covid-19 vaccine? |  |  |  |  |  |
| 314 | Newly discovered COVID-19 vaccine that is given for Ethiopia is the actual one that the discovered country is using yet |  |  |  |  |  |

**Part IV: Question related to perception towards COVID-19 vaccine**

| **Code** | **Variable** | **Response** |
| --- | --- | --- |
| **401** | Have you ever been infected with COVID-19? | _1_ Yes _2_ No _3_ I don’t know |
| **402** | Do you think that the COVID-19 vaccine is effective? | _1_ Yes _2_ No _3_ I don’t know |
| **403** | Do you think that COVID-19 vaccination is mandatory for health care workers | _1_ Yes _2_ No _3_ I don’t know |
| **404** | Do you think the newly discovered COVID-19 vaccine may have side effects? | _1_ Yes _2_ No _3_ I don’t know |
| **405** | Do you think that if everyone in the society maintains the preventive measures, the COVID-19 pandemic can be eradicated without vaccination? | _1_ Yes _2_ No _3_ I don’t know |
| **406** | Are you aware that there are several candidate COVID-19 vaccines being developed? | _1_ Yes _2_ No _3_ I don’t know |
| **407** | Do you think the COVID-19 vaccine will be affordable and accessible by the common man? | _1_ Yes _2_ No _3_ I don’t know |

**Thank you!**
