# Supplementary material for: Attitude of health professionals towards COVID-19 vaccination and associated factors among health professionals, Western Ethiopia: A cross-sectional survey
Source: PLoS One. 2022 Mar 9;17(3):e0265061. doi: 10.1371/journal.pone.0265061 (PMC8906598; doi:10.1371/journal.pone.0265061)
Supplement: Attachment — Submitted filename: PONE-D-21-21414.pdf [file pone.0265061.s005.pdf]

PLOS ONE

Attitude and perception of health professionals towards COVID-19 vaccination and associated factors among health professionals found in Health facilities of Nekemte town, Western Ethiopia

--Manuscript Draft--

**Manuscript Number:**	PONE-D-21-21414
**Article Type:**	Research Article
**Full Title:**	Attitude and perception of health professionals towards COVID-19 vaccination and associated factors among health professionals found in Health facilities of Nekemte town, Western Ethiopia
**Short Title:**	Attitude and perception of health professionals towards COVID-19 vaccination and associated factors
**Corresponding Author:**	Tadesse Tolossa, MPH<br>Wollega University<br>Nekemte, Oromia ETHIOPIA
**Keywords:**	Attitude, COVID-19, Health professionals, vaccination, Ethiopia
**Abstract:**	Introduction: Even though people of the world were eagerly waiting for the hope of vaccine development, vaccine hesitancy is becoming the top concern in both developed and developing countries. However, there is no adequate evidence regarding the attitude and perception of health professionals towards COVID 19 vaccine in resource limited settings like Ethiopia. Hence, this study was aimed to assess the attitude and perception of health professionals towards COVID 19 vaccine in West Ethiopia.<br>Methods: An institution-based cross-sectional study was conducted among health care workers found in Nekemte town from April 14-21, 2021. A total of 439 health professionals present on duty during study period was included in the study. The data were collected by using self-administered questionnaire. Epidata version 3.2 was used for data entry, and STATA version 14 was used for data analysis. The binary logistic regression model was employed to determine factors associated with the attitude towards COVID-19 vaccination. Adjusted Odds Ratio (AOR) with 95% confidence intervals was computed and statistical significance was declared at a 5% level (p-value < 0.05).<br>Result: A total of 431 health professionals were participated in the study yielding a response rate of 98.1%. The study indicate that 51.28% (95%CI: 45.12%, 57.34%) of health professionals had favorable attitude towards COVID-19 vaccination. Having good knowledge about COVID-19 vaccine (AOR=0.38, 95%CI: 0.22, 0.64) was the negatively associated with unfavorable attitude towards COVID-19 vaccine, whereas working in private clinic (AOR=7.77, 95% CI: 2.19, 27.58) and health center (AOR=2.45, 95%CI: 1.01, 5.92) were positively associated with the of unfavorable attitude towards COVID-19 vaccine.<br>Conclusion and recommendation: In general, the attitude and perception of health care professionals toward COVID-19 vaccine in the study area was unsatisfactory. Having poor knowledge about COVID-19 vaccine, working in private clinic and health center are the independent determinants of unfavorable attitude towards COVID-19 vaccine. Therefore, there is still a need to improve the knowledge of health professionals toward COVID-19 vaccine through providing reliable information regarding the vaccine safety, efficacy and effectiveness.
**Order of Authors:**	Tadesse Tolossa, MPH
	Bizuneh Wakuma
	Ebisa Turi
	Diriba Mulisa
	Diriba Ayala
	Getahun Fetensa

Powered by Editorial Manager® and ProduXion Manager® from Aries Systems Corporation

	Belayneh Mengist
	Gebeyehu Abera
	Emiru Merdassa
	Dejene Seyoum
	Tesfaye Shibiru
**Additional Information:**	
**Question**	**Response**
**Financial Disclosure**<br><br>Enter a financial disclosure statement that describes the sources of funding for the work included in this submission. Review the submission guidelines for detailed requirements. View published research articles from *PLOS ONE* for specific examples.<br><br>This statement is required for submission and **will appear in the published article** if the submission is accepted. Please make sure it is accurate.<br><br>**Unfunded studies**<br>Enter: *The author(s) received no specific funding for this work.*<br><br>**Funded studies**<br>Enter a statement with the following details:<br>• Initials of the authors who received each award<br>• Grant numbers awarded to each author<br>• The full name of each funder<br>• URL of each funder website<br>• Did the sponsors or funders play any role in the study design, data collection and analysis, decision to publish, or preparation of the manuscript?<br>• **NO** - Include this sentence at the end of your statement: *The funders had no role in study design, data collection and analysis, decision to publish, or preparation of the manuscript.*<br>• **YES** - Specify the role(s) played.<br><br>* typeset	The author(s) received no specific funding for this work
**Competing Interests**<br><br>Use the instructions below to enter a competing interest statement for this submission. On behalf of all authors,	The authors have declared that no competing interests exist.

disclose any competing interests that could be perceived to bias this work—acknowledging all financial support and any other relevant financial or non-financial competing interests.<br><br>This statement is required for submission and **will appear in the published article** if the submission is accepted. Please make sure it is accurate and that any funding sources listed in your Funding Information later in the submission form are also declared in your Financial Disclosure statement.<br><br>View published research articles from *PLOS ONE* for specific examples.<br><br>**NO authors have competing interests**<br><br>Enter: *The authors have declared that no competing interests exist.*<br><br>**Authors with competing interests**<br><br>Enter competing interest details beginning with this statement:<br><br>*I have read the journal's policy and the authors of this manuscript have the following competing interests: [insert competing interests here]*<br><br>* typeset	
**Ethics Statement**<br><br>Enter an ethics statement for this submission. This statement is required if the study involved:<br><br>• Human participants<br>• Human specimens or tissue<br>• Vertebrate animals or cephalopods<br>• Vertebrate embryos or tissues<br>• Field research<br><br>Write "N/A" if the submission does not require an ethics statement.	N/A

Powered by Editorial Manager® and ProduXion Manager® from Aries Systems Corporation

General guidance is provided below. Consult the submission guidelines for detailed instructions. **Make sure that all information entered here is included in the Methods section of the manuscript.**<br><br>**Format for specific study types**<br><br>**Human Subject Research (involving human participants and/or tissue)**<br>• Give the name of the institutional review board or ethics committee that approved the study<br>• Include the approval number and/or a statement indicating approval of this research<br>• Indicate the form of consent obtained (written/oral) or the reason that consent was not obtained (e.g. the data were analyzed anonymously)<br><br>**Animal Research (involving vertebrate animals, embryos or tissues)**<br>• Provide the name of the Institutional Animal Care and Use Committee (IACUC) or other relevant ethics board that reviewed the study protocol, and indicate whether they approved this research or granted a formal waiver of ethical approval<br>• Include an approval number if one was obtained<br>• If the study involved *non-human primates*, add *additional details* about animal welfare and steps taken to ameliorate suffering<br>• If anesthesia, euthanasia, or any kind of animal sacrifice is part of the study, include briefly which substances and/or methods were applied<br><br>**Field Research**<br><br>Include the following details if this study involves the collection of plant, animal, or other materials from a natural setting:<br>• Field permit number<br>• Name of the institution or relevant body that granted permission	
**Data Availability**<br><br>Authors are required to make all data	Yes - all data are fully available without restriction

Powered by Editorial Manager® and ProduXion Manager® from Aries Systems Corporation

underlying the findings described fully available, without restriction, and from the time of publication. PLOS allows rare exceptions to address legal and ethical concerns. See the PLOS Data Policy and FAQ for detailed information.<br><br>A Data Availability Statement describing where the data can be found is required at submission. Your answers to this question constitute the Data Availability Statement and **will be published in the article**, if accepted.<br><br>**Important:** Stating 'data available on request from the author' is not sufficient. If your data are only available upon request, select 'No' for the first question and explain your exceptional situation in the text box.<br><br>Do the authors confirm that all data underlying the findings described in their manuscript are fully available without restriction?	
**Describe where the data may be found in full sentences. If you are copying our sample text, replace any instances of XXX with the appropriate details.**<br><br>• If the data are **held or will be held in a public repository**, include URLs, accession numbers or DOIs. If this information will only be available after acceptance, indicate this by ticking the box below. For example: *All XXX files are available from the XXX database (accession number(s) XXX, XXX.*).<br>• If the data are all contained **within the manuscript and/or Supporting Information files**, enter the following: *All relevant data are within the manuscript and its Supporting Information files.*<br>• If neither of these applies but you are able to provide **details of access elsewhere**, with or without limitations, please do so. For example:<br><br>*Data cannot be shared publicly because of [XXX]. Data are available from the*	All relevant data are within the manuscript and its Supporting Information files.

XXX Institutional Data Access / Ethics Committee (contact via XXX) for researchers who meet the criteria for access to confidential data.<br><br>*The data underlying the results presented in the study are available from (include the name of the third party and contact information or URL).*<br>• This text is appropriate if the data are owned by a third party and authors do not have permission to share the data.<br><br>* typeset	
Additional data availability information:	

manuscript Click here to access/download;Manuscript;manuscript.docx

Attitude and perception of health professionals towards COVID-19 vaccination and associated factors among health professionals found in Health facilities of Nekemte town, Western Ethiopia

Tadesse Tolossa[1*], Bizuneh Wakuma[2], Ebisa Turi[1], Diriba Mulisa[2], Diriba Ayala[3], Getahun Fetensa[2], Belayneh Mengist[4], Gebeyehu Abera[5], Emiru Merdassa Atomssa[1], Dejene Seyoum[1], Tesfaye Shibiru[6]

[*1]Department of Public Health, Institute of Health Science, Wollega University, Nekemte Ethiopia

[2]Department of Nursing, Institute of Health Science, Wollega University, Nekemte Ethiopia

[3]Department of Midwifery, Institute of Health Science, Wollega University, Nekemte, Ethiopia

[4]Department of Public Health, College of Health Science, Debre Markos University, Debre Markos, Ethiopia

[5]Nekemte Health Center, Nekemte, Ethiopia

[6] School of Medicine, Institute of Health Sciences, Wollega University, Nekmete Ethiopia

Corresponding author: **Tadesse Tolossa[1*]**

[1] Department of Public Health, Institutes of Health Sciences, Wollega University, Nekemte, Ethiopia*

E-mail- yadanotolasa@gmail.com

P.O.BOX: 395

Nekemte, Ethiopia

Tel: +251911936804

Abstract

Introduction: Even though people of the world were eagerly waiting for the hope of vaccine development, vaccine hesitancy is becoming the top concern in both developed and developing countries. However, there is no adequate evidence regarding the attitude and perception of health professionals towards COVID 19 vaccine in resource limited settings like Ethiopia. Hence, this study was aimed to assess the attitude and perception of health professionals towards COVID 19 vaccine in West Ethiopia.

Methods: An institution-based cross-sectional study was conducted among health care workers found in Nekemte town from April 14-21, 2021. A total of 439 health professionals present on duty during study period was included in the study. The data were collected by using self-administered questionnaire. Epidata version 3.2 was used for data entry, and STATA version 14 was used for data analysis. The binary logistic regression model was employed to determine factors associated with the attitude towards COVID-19 vaccination. Adjusted Odds Ratio (AOR) with 95% confidence intervals was computed and statistical significance was declared at a 5% level (p-value < 0.05).

Result: A total of 431 health professionals were participated in the study yielding a response rate of 98.1%. The study indicate that 51.28% (95%CI: 45.12%, 57.34%) of health professionals had favorable attitude towards COVID-19 vaccination. Having good knowledge about COVID-19 vaccine (AOR=0.38, 95%CI: 0.22, 0.64) was negatively associated with unfavorable attitude towards COVID-19 vaccine, whereas working in private clinic (AOR=7.77, 95% CI: 2.19, 27.58) and health center (AOR=2.45, 95%CI: 1.01, 5.92) were positively associated with the of unfavorable attitude towards COVID-19 vaccine.

Conclusion and recommendation: In general, the attitude and perception of health care professionals toward COVID-19 vaccine in the study area was unsatisfactory. Having [illegible]r knowledge about COVID-19 vaccine, working in private clinic and health center are the independent determinants of unfavorable attitude towards COVID-19 vaccine. Therefore, there is still a need to improve the knowledge of health professionals toward COVID-19 vaccine through providing reliable information regarding the vaccine safety, efficacy and effectivenes[illegible]

Keywords: Attitude, COVID-19, Health professionals, vaccination, Ethiopia

Introduction

Starting from the day it was declared as pandemic, the COVID-19 remains the worst Global public health challenges. According to worldometer report the coronavirus COVID-19 is affecting about 220 countries and territories. More than 170 million cases, and 3.5 million deaths happened due to COVID-19 (1, 2). Based to this, the pandemic brought the double burden in developing countries which are already overwhelmed by the health care system challenges (3).

Given the sub-Saharan Africans are not the highest shareholder by cases and death during the early phase(4), the direct of COVID-19 and the indirect effect of its mitigation disrupted the health care services (5). The serious of preventive measures (movement restriction, physical distancing, lockdowns, hand washing and sanitizing) were practiced (6). However, the counter effect of some mitigation brought significant change in health care settings especially by task shifting and task sharing. Its effect on the economy is also evident. Though these mitigation procedures played paramount role in averting burden of COVID19, the whole world were hoping for the vaccine development (7).

Around the end of 2020, quite a lot of vaccines started to arise; there are about hundreds of candidate vaccines (7). As of June 10,2021, from 287 candidate vaccines, 102 are in clinical phase, 185 are in pre-clinical phase (8). From these, WHO has listed the Pfizer/BioNTech, Astrazeneca-SK Bio, Serum Institute of India, Janssen and Moderna vaccines for emergency use (6, 7). Believing H[illegible] are explicitly exposed to increased risk of infection through direct contact with patients, they are supposed to prioritized for vaccination (9).

Willingness of the general population to accept the vaccine ahead of COVID-19 vaccine development was relatively promising when compared to today's reality (10-12). Even though people of the world were eagerly waiting for the hope of vaccine development, vaccine hesitancy is becoming the top concern in both developed and developing countries (11, 13-17). For instance, the Vaccine acceptance is 21% in Egy[illegible]18), 54.6% in China(19), and 57.6% of adult population in US(20) intended to be vaccinated. The most commonly reason mentioned for hesitancy were concerns about safety of a vaccine and wide-ranging lack of confidence, worries about the efficiency of the vaccine(17, 18, 21)

Several evidences indicate that healthcare professionals (HCPs) play a paramount role in this regard and can significantly affect the general public's decisions to receive the COVID- 19 vaccine(22, 23). In Ethiopia, there have been 169,640 confirmed cases of COVID-19, with 9651 deaths till now May 30, 2021 (1). The Ethiopia government, and Ethiopian Ministry of health exerts great efforts to provide the COVID-19 vaccines and sort the vaccination as a priority for healthcare workers (HCWs) and older people, especially with chronic diseases (24). To overcome the expected upcoming challenge of vaccination hesitancy, we have to measure and know the exact reasons behind it. However, there is no adequate evidence regarding the attitude and perception of health professionals towards COVID 19 vaccine in resource limited settings like Ethiopia. Hence, this study was aimed to assess the attitude and perception of health professionals towards COVID 19 vaccine in West Ethiopia.

Methods

Study area and period

This study was conducted in health institutions found in Nekemte town. Nekmete is a capital city of East Wollega Zone, and it is located 330 KM from Addis Ababa the capital city of Ethiop[illegible] The study was conducted from April 15-21, 2021. Data from Nekemte Town Health Office showed that, the town has one teaching referral hospital, which is owned by Wollega University, one specialized hospital administered under Oromia regional Health bureau, two health centers (Nekemte and Cheleleki Health center), and more than fifteen medium and above private clinics. More than 800 health professionals are found in Nekemte town.

Study design

Institutional based cross sectional study design was employed.

Population, sample size and sampling techniques

All Health professionals working [illegible]king in private and public health institutions of Nekemte town were a source population. Health professionals who were not on duty due to different reason were excluded from the study. All health professionals who were on duty during data collection, and have willingness to participate in study were selected for study.

Sample size was determined by single population proportion with the following assumptions: Since this study was the first of its type in Ethiopia P=50% was taken, with 5% margin of error, and 95% confidence level. Accordingly the calculated sample size was 384 and after adding a 15% allowance for a non-response rate; the final sample size was = **439** health professiona[illegible].

Regarding the sampling techniques, all health institutions found in Nekemte town were included in the study. There are two public hospitals in Nekemte town, two health centers, and 15 medium clinics. Then the sample size was proportionally allocated to hospital, health center and private clinics. Around 710 health professionals are working in two hospitals, 50 in two health centers and 60 health professionals in all private clinics. For hospitals, we have used the identification number of the health professionals and the identification number was used as a sampling frame. Then computer generated simple random sampling technique was used to select the sample. Health professionals who were not on duty during study period were excluded from the sampling fram[illegible]For health center and private clinic, all health professionals who available during study period were included in the study. Furthermore, health professionals working in more than one health facilities were considered only in a single health facilities to prevent any distortion of information.

Variables

Attitude of health professionals towards COVID-19 vaccine was the dependent variable of this study. Ten items were used to assess the attitu[illegible]ealth professionals towards COVID-19 cases. Each item was measured on likert-scale with a five points scale (strongly agree, agree, neutral, disagree, strongly disagree) to allow the study participants to express how much they agree or disagree with a particular question. “Favorable attitu[illegible] was when the scoring was ≥ mean score and less than mean value was rated as an “unfavorable attitude” towards COVID-19 vaccine. Socio demographic variables suc[illegible]age, sex, marital status, educational level, educational background, religion), medical history (chronic medical disease and previously infected with COVID-19), knowledge towards COVID-19 vaccine, and perception towards COVID-19 vaccine) were independent variables of this study. The knowledge of health professionals towards COVID-19 vaccine was assessed by 5 items. Each items contained the category of “Yes” and “No”. A correct answer was assigned “1” point and an incorrect answer was assigned “0” points. The total score ranged from 0 to 5. “Good knowledge” was when the scoring was ≥

mean score and score below mean value indicated "poor knowledge" towards COVID-19 vaccine.

Finally, the perception of participants towards COVID-19 vaccine was assessed by using five items with "Yes"/No" category. Respondents who scored greater than or equal the mean score were grouped to have "good perception" and participants who scored less than mean score were grouped to have "poor perception" towards COVID-19 vaccin[illegible]

Data collection techniques and data quality assurance

A questionnaire was developed by reviewing previously published papers (25, 26). The questionnaires comprised of socio-demographic data, medical history, knowledge, attitude, and perception towards COVID-19 vaccine. The data was collected by using self-administered questionnaires. The questionnaire was given to all health professionals who were present on duty, and returned back to data collectors after they filled it. The questionnaire was prepared in English, and the content of the questionnaire was explained for them prior to distributing it.

The questionnaire was pre-tested on 5% of participants. Then possible amendments were done based on the findings. Discussion was held between investigators and data collectors, based on the result of the pre-test and accordingly, some amendments were made. One day training was given to the data collectors about the tool and data collection procedure. Data were checked for completeness, accuracy, clarity, and consistency by the supervisors and principal investigator on daily basis. Any error or ambiguity and incompleteness were corrected accordingly.

Data management and analysis

Epidata version 3.0 was used for data entry, and exported to STATA version 14.0 for further analysis. Descriptive statistics, like frequencies, percentages, mean and standard deviation were computed. Before analysis, data were cleaned and edited by using simple frequencies and cross-tabulation. Re-categorization of categorical variables and categorization of continuous variables was done. The assumption of the logistic regression model was checked before fitting to the model. The binary logistic regression model was fitted to determine factors associated with COVID-19 vaccine acce[illegible]bility. Factors that were associated with the outcome variable at 20 % (P-value $\leq$0.20) significant level in the bivariable logistic regression analysis were included in the multivariable logistic regression analysis. Then crude and adjusted odds ratio together with

their corresponding 95% confidence intervals were presented in the final multivariable logistic regression table. [illegible]R with 95% confidence intervals was computed and statistical significance was declared when it was significant at a 5% level (p-value < 0.05). Multicollinearity (association between explanatory variables) was checked by correlation matrices, and the model goodness of fit test was checked by Hosmer and Lomeshow test.

Ethical considerations

The study was approved and ethical clearance letters was obtained from Wollega University, Institute of Health Science research Ethics review board. After approval, permission letter was obtained from administrative body of health facilities to respective clinics. A verbal consent was obtained from study participants and the purpose of this study was stated to all participants. Everybody was participated voluntarily in this study.

Results

Socio demographic characteristics of the health professionals

A total of 431 health professionals were interviewed and yielded a response rate of 98.1%. More than half of the total participants 268 (62.28%) were male. Regarding to the age of the health professionals nearly two-third of them 274 (63.57 %) belongs to less than 30 years old age group. More than half 253(58.07%) of the participants were protestant religion followers. Greater than three fourth of the health professionals participated in this study were from hospital institutions 350 (81.21%) (Table 1)

Table 1: **Socio demographic characteristics of the health professionals in Nekemte City, Western Ethiopia, 2021**

**Variables**	**Option**	**Frequency**	**Percent**
Age	<[illegible]ears old	274	63.57
	>=30years old	157	36.43
Sex	Male	268	62.18
	Female	163	37.82
Marital status	Never married	176	40.84
	Married	243	56.38
	Separated	8	1.86
	Others	4	0.93
Religion	Muslim	73	16.94
	Protestant	253	58.70
	Catholic	10	2.32

	Orthodox	71	16.47
	Others	24	5.57
Institution	Hospitals	350	81.21
	Health center	34	7.89
	Private	47	10.90
Education level	Diploma	38	8.82
	First degree	362	83.99
	Masters	24	5.57
	Doctors	2	0.46
	Specialty degree	5	1.16
Back ground	Nurses	186	43.16
	Midwifery	56	12.99
	Pharmacy	32	7.42
	Medical practitioner	47	10.90
	Public health	41	9.51
	Anesthetists	19	4.41
	MLS	36	8.35
	Psychiatrics	6	1.39
	others	8	1.86

Medical disorder related characteristics of the health professionals

Thirty three health care workers have history of taking vaccination in their life 33 (7.66%). [illegible]s than quarter of the health care workers in this study have chronic diseases 19 (4.41%). The most commonly reported chronic disease by participants was hypertension 6 (46.15%). Again in this study greater than half of the participants were vaccinated against corona virus 240 (55.68%).

Attitude of the health professionals regarding vaccination against corona virus

In this study, almost ha[illegible]f the participants have poor attitude for corona virus vaccination 210 (48.72 %). One-third of the participants strongly agreed that COVID-19 cannot be controlled without vaccination. Moreover, nearly one-third of participants agreed that mass vaccination against corona virus helps to overcome the pandemic Regarding to effectiveness of the newly discovered corona virus vaccination, less than quarter of the participants are strongly disagree 63 (14.62%)(**Figure 1**).

Table 2: Attitude of the health care workers regarding vaccination against corona viru[illegible] 2021

Variables	Option	Frequency	Percent
Vaccine that is currently given in Ethiopia is actual one that those innovated country are taking	Strongly disagree	114	26.45
	Disagree	132	30.63
	Neutral	81	18.79
	Agree	79	18.33
	Strongly agree	25	5.80
If one person take corona virus vaccination it has great contribution for other people	Strongly disagree	65	15.08
	Disagree	86	19.95
	Neutral	53	12.30
	Agree	155	35.96
	Strongly agree	72	16.71
I will take the vaccine if I get without hesitation	Strongly disagree	61	14.15
	Disagree	183	42.46
	Neutral	51	11.83
	Agree	86	19.95
	Strongly agree	50	11.60
I encourage my family and others to take the vaccination	Strongly disagree	58	13.46
	Disagree	135	31.32
	Neutral	61	14.15
	Agree	127	29.47
	Strongly agree	50	11.60
Covid-19 cannot be controlled without vaccination	Strongly disagree	85	19.72
	Disagree	136	31.55
	Neutral	65	15.08
	Agree	111	25.75
	Strongly agree	34	34
Corona virus vaccine is fairly distributed for all	Strongly disagree	67	15.55
	Disagree	122	28.31
	Neutral	84	19.49
	Agree	99	22.97
	Strongly agree	59	13.69
Mass vaccination can overcome the epidemic attach of the corona virus	Strongly disagree	53	12.30
	Disagree	90	20.88
	Neutral	77	17.87
	Agree	138	32.02
	Strongly agree	73	16.94
The best prevention method is to take vaccine against corona virus	Strongly disagree	54	12.53
	Disagree	91	21.11
	Neutral	92	21.35
	Agree	134	31.09
	Strongly agree	60	13.92
The corona virus vaccine is not tested adequately	Strongly disagree	28	6.50

for its effectiveness	Disagree	73	16.94
	Neutral	67	15.55
	Agree	129	29.93
	Strongly agree	134	31.09
After vaccinated against corona virus other precaution can be avoided	Strongly disagree	89	20.65
	Disagree	131	30.39
	Neutral	62	14.39
	Agree	66	15.31
	Strongly agree	83	19.26
Overall attitude towards vaccine against corona virus	Favorable	221	51.28
	Unfavorable	210	48.72

Perceptio[illegible]he health care workers regarding Corona virus vaccine

The distributions of each perception item about COVID-19 vaccine are presented in Table 3. With regard to the question ‘Do you think the corona virus vaccine is effective’? Nearly one-third of them thought the vaccine against corona virus is effective. More than half of the participants responded unsure for the question ‘Do you think COVID-19 will be controlled only by preventive measures without vaccination’? (Table 3). Almost half of the participants accept that newly discovered Corona virus vaccine has side effect 209 (48.49%) (Figure 2)

Table 3: Perception the health care workers regarding Corona virus vaccine, 2021

Variables	Option	Frequency	Percent
Have you ever infected with corona virus	Yes	95	22.04
	No	169	39.21
	I don’t know	167	38.75
Do you think that corona virus vaccine is effective?	Yes	131	30.39
	No	34	7.89
	I don’t know	266	61.72
Do you think that covid vaccine is mandatory for health care worker?	Yes	262	60.79
	No	262	60.79
	I don’t know	145	33.64
Do you think COVID-19 will be controlled only by preventive measures without vaccination?	Yes	85	19.72
	No	102	23.67
	I don’t know	244	56.61
Do you think that there are various corona virus vaccine that have discovered?	Yes	142	32.95
	No	46	10.67
	I don’t know	243	56.38
Do you think that covid-19 vaccine will be affordable and accessible by the common man!	Yes	59	13.69
	No	145	33.64

	I don’t know	227	52.67

Factors associated with Attitude of health professionals toward corona virus vaccine.

In multivariable logistic regression, variables like age, sex, institution types, having chronic diseases and allergic reaction to previous medication have shown significant association with the outcome of interest. From those variables, two of them showed significant association with attitude of health professionals toward vaccine against corona virus. The likelihood of having poor attitude toward COVID-19 vaccine was 2.45 times higher among Health professionals who are working at health center compared to those working at hospitals AOR=2.45(95%CI 1.01,5.92). Similarly, the odds of having poor attitude towards COVID-19 vaccine was 7.77 times higher among health professionals working at private clinic those working at the hospitals AOR=7.77(95% CI 2.19,27.58). Moreover, the proportion of health professionals having unfavorable attitude was 62% lower among professionals who have good knowledge toward COVID-19 vaccination than their counterparts AOR=0.38(95%CI: 0.22, 0.6[illegible] Table 4).

Table 4: Multivariable analysis of factors associated with attitude towards COVID-19 vaccine among health professionals in Nekemte City, Western Ethiopia, 2021

Characteristics		Attitude		COR(95%CI)	AOR(95%CI)	P-value
		Good	[illegible]r			
Age	<30	125	149	1.87(1.25,2.79)	2.14(1.25,3.67)	<0.01
	>=30	96	61	Ref		
Sex	Male	143	125	Ref		
	Female	78	85	0.80 (0.54,1.18)	0.93(0.54, 1.59)	0.795
Institution	Hospital	155	195	Ref		
	Health center	24	10	3.01(1.40, 6.50)	2.45(1.01,5.92)	0.045*
	Private clinic	42	5	10.56 (4.0,27.35)	7.77(2.19,27.58)	<0.01*
Chronic diseases	Yes	10	9	Ref		
	No	211	201	0.94(0.37,2.37)	0.76(0.25,2.29)	0.630
History of allergic reaction to previous medication	Yes	20	21	Ref		
	No	201	189	1.11(0.58,2.12)	1.04(0.46,2.33)	0.914
Knowledge about Corona virus vaccination	Poor knowledge	86	44	Ref		
	Good knowledge	163	139	0.30(0.18,0.49)	0.38(0.22,0.64)	<0.01*

Discussion

Though the vaccine development against COVID-19 was promising for the world population, vaccine hesitancy has become a global challenge to the successful uptake of the vaccine (11, 13-17). Literatures are reporting as vaccine safety and efficacy are among the worries of the peoples and the reasons for vaccine hesitancy (17, 18, 21). Health care professionals are at a particular concern to be vaccinated so that they can positively influence the general population for vaccination. With this regard there was dearth of information about the attitude and perception of health professionals toward COVID-19 vaccination and its associated factor in Ethiopia, particularly in the study area. Therefore, this study was intended to determine Attitude and perception of health professionals toward COVID-19 vaccination and its associated factors in Nekemte town.

Accordingly in the present study, 31[illegible]% of the study participants had agreed to take the vaccine without hesitation if available. This is lower than the study finding from Bangladesh (58.6%)(27), Canada (72.40)(28). The possible explanation for this might be the variation in trust and reliability of the source of information about COVID-19 vaccine. Moreover, 41.07% of the study subjects in the present study have also agreed on encouraging family and others to take the vaccine while 65.5% of participants in Bangladesh agreed to do so. Furthermore, 59.75% of health professionals in this study agreed on COVID-19 won't be controlled without vaccination. This echoes the finding from Bangladesh (63.4%)(27). In addition, nearly half of the study participants in the current study have agreed on mass vaccination to overcome the pandemic. This is also congruent with the study finding reported by Elhad[illegible]M et al in Libya(29). Comparable to the study finding from Saudi Arabia (37%)(30), in the present study, only one third of participants agreed on the effectiveness of the vaccine. This depicts that there is still a need to provide reliable and accurate information about effectiveness of the vaccine against COVID-19 to the health care professionals and the public in gener[illegible].

The current study revealed that half of the health care professionals participated in this study have favorable attitude toward COVID-19 vaccination. This is lower than the study done in Bangladesh that reported 78% of the general population had favorable attitude(27). However, it is higher than the online survey done in Ethiopia that reported 24.2% of the general population had favorable attitude toward COVID-19 vaccination(31). The possible reason for the observed

discrepancy might be due to the relative information difference among the population of interest. Furthermore, it might be due to health care professionals are at higher risk of infection with the virus that might result in having favorable attitude toward vaccination in order to minimize risk of infection. This implies there is a need to clearly assure the effectiveness, safety and efficacy of the vaccine to enhance its uptake by health professionals and the general public. It is clear that health professionals are the counselor and advocators of the general population that their decision to receive the vaccine can greatly influences the uptake of the vaccine by the general population.

With regard to the factors affecting the attitude of health care professionals toward COVID-19 vaccination, knowledge toward COVID-19 vaccination and type of health facility were independent determinants. Accordingly, the proportion of health professionals having unfavorable attitude was 62% lower among professionals who have good knowledge toward COVID-19 vaccination than their counterparts AOR=0.38(95%CI: 0.22, 0.64)). This might be due to the fact that knowing the importance of corona virus vaccine can positively influence someone to have positive attitude toward it. Moreover, the odds of having unfavorable attitude was much higher among health professionals working in private clinic and health center than those in Hospital. This variation might be due to the relative number of client flow to the health center and private clinic is much lower than the hospital and, hence the professionals consider themselves as at lower risk of exposure and infection to COVID-19. In addition, almost all COVID-19 patients have been admitted to hospitals than private clinic or health center. Therefore, professionals working at health center and private clinics for known and obvious reason consider themselves as having lower risk of infection with corona virus, and as a result they might have unfavorable attitude toward COVID-19 vaccine than professionals working at hospital settin[illegible]

Limitation of the study

The study did not provide the qualitative perspectives on the attitude and perception of health professionals towards COVID-19 vaccine and its associated factors, which if available, could underpin the quantitative findings. Moreover, since it is cross sectional study it did not addressed the cause and effect relationship of the factors and the outcome variables.

Conclusion

In general, the attitude and perception of health care professionals toward COVID-19 vaccine in the study area was unsatisfactory. Having poor knowledge about COVID-19 vaccine, working in private clinic and health center are the independent determinants of unfavorable attitude towards COVID-19 vaccine. Therefore, there is still a need to improve the knowledge of health professionals toward COVID-19 vaccine through providing reliable information regarding the vaccine safety, efficacy and effectiveness. Furthermore, the media outlets need to work to develop trust among the public by disseminating accurate and consistent information about the vaccine. In addition, the future researchers should also explore more about the attitude and perception of health care professionals toward COVID-19 vaccine and its determinants using qualitative data.

Abbreviations

AOR: Adjusted Odds Ratio: **COR**: Crude Odds Ratio, **HCW**: Health Care Worker, **MLS**: Medical Laboratory Science, **WURH**: Wollega University Referral Hospital

Declaration

Ethical approval and consent to participate

The study was approved and ethical clearance letters was obtained from Wollega University, Institute of Health Science research Ethics review board. After approval, permission letter was obtained from administrative body of each health facility. A verbal consent was obtained from study participants and the purpose of this study was stated to all participants. Everybody was participated voluntarily in this study.

Consent to publish

Not applicable

Availability of Data and Materials

All data analyzed during this study are included in the manuscript.

Competing Interests

The authors declare that they have no competing interests.

Funding

No funding was obtained for this study.

Authors' Contribution

TT, BW, ET, GF, BM, DA, GA, and EM involved in the initial development of proposal. TT involved in statistical analysis. TT, TS, and DS involved in developing the initial drafts of the manuscript. All authors participated in the final preparation of manuscript and they approved the final draft of the manuscript for submission.

Acknowledgments

We would like to thank all health facilities for their invaluable co-operation during data collection and our deep acknowledge also goes to the data collectors for their interest and commitment in carrying out the study.

Author Information

[*1]Department of Public Health, Institute of Health Science, Wollega University, Nekemte Ethiopia

[2]Department of Nursing, Institute of Health Science, Wollega University, Nekemte Ethiopia

[3]Department of Midwifery, Institute of Health Science, Wollega University, Nekemte, Ethiopia

[4]Department of Public Health, College of Health Science, Debre Markos University, Debre Markos, Ethiopia

[5]Nekemte Health Center, Nekemte, Ethiopia

[6] School of Medicine, Institute of Health Sciences, Wollega University, Nekmete Ethiopia

References

1. Worldometer. COVID-19 Coronavirus Pandemic. Available online: https://www.worldometers.info/coronavirus/?utmcampaign=homeAdvegas1? (accessed on May 30, 2021). 2021.
2. Du R-H, Liang L-R, Yang C-Q, Wang W, Cao T-Z, Li M, et al. Predictors of mortality for patients with COVID-19 pneumonia caused by SARS-CoV-2: a prospective cohort study. European Respiratory Journal. 2020;55(5).
3. Wachamo D, Tegene Y, Tibeso A, Washo A. Sexual and Reproductive Health Services Utilization and Associated Factors among College Students at West Arsi Zone in Oromia Region, Ethiopia. The Scientific World Journal. 2020;2020.
4. Mehtar S, Preiser W, Lakhe NA, Bousso A, TamFum J-JM, Kallay O, et al. Limiting the spread of COVID-19 in Africa: one size mitigation strategies do not fit all countries. The Lancet Global Health. 2020;8(7):e881-e3.
5. Muhaidat N, Fram K, Thekrallah F, Qatawneh A, Al-Btoush Aa. Pregnancy During COVID-19 Outbreak: The Impact of Lockdown in a Middle-Income Country on Antenatal Healthcare and Wellbeing. International Journal of Women's Health 2020;12:1065–73
6. Mosisa G, Tsegaye R, Wakuma B, Mulisa D, Etefa W, Abadiga M, et al. Fertility desire and associated factors among people living with HIV in Ethiopia: a systematic review and meta-analysis. Archives of Public Health. 2020;78(1):1-16.
7. Jasim SK. Post Cesarean Section Surgical Site Infection; Incidence and Risk Factors.
8. World Health Organization COVID-19 vaccine tracker and landscape: https://www.who.int/publications/m/item/draft-landscape-of-covid-19-candidate-vaccines. 2021.
9. Duch R, Roope LS, Violato M, Becerra MF, Robinson T, Bonnefon J-F, et al. Who should be first in line for the COVID-19 vaccine? Surveys in 13 countries of the publics preferences for prioritisation. medRxiv. 2021.
10. Belsti Y, Gela YY, Akalu Y, Dagnew B, Getnet M, Seid MA, et al. Willingness of Ethiopian Population to Receive COVID-19 Vaccine. Journal of Multidisciplinary Healthcare. 2021;14:1233.
11. Kaplan AK, Sahin MK, Parildar H, Guvenc IA. The willingness to accept the COVID-19 vaccine and affecting factors among healthcare professionals: A cross-sectional study in Turkey. International Journal of Clinical Practice. 2021:e14226.
12. Dodd RH, Cvejic E, Bonner C, Pickles K, McCaffery KJ, Ayre J, et al. Willingness to vaccinate against COVID-19 in Australia. The Lancet Infectious Diseases. 2021;21(3):318-9.
13. Alabdulla M, Reagu SM, Al-Khal A, Elzain M, Jones RM. COVID-19 vaccine hesitancy and attitudes in Qatar: A national cross-sectional survey of a migrant-majority population. Influenza and other respiratory viruses. 2021;15(3):361-70.
14. Khubchandani J, Sharma S, Price JH, Wiblishauser MJ, Sharma M, Webb FJ. COVID-19 vaccination hesitancy in the United States: a rapid national assessment. Journal of Community Health. 2021;46(2):270-7.
15. Lake EA, Demissie BW, Gebeyehu NA, Wassie AY, Gelaw KA, Azeze GA. Knowledge, attitude and practice towards COVID-19 among health professionals in Ethiopia: A systematic review and meta-analysis. PloS one. 2021;16(2):e0247204.
16. Latkin CA, Dayton L, Yi G, Konstantopoulos A, Boodram B. Trust in a COVID-19 vaccine in the US: A social-ecological perspective. Social science & medicine (1982). 2021;270:113684.
17. Troiano G, Nardi A. Vaccine hesitancy in the era of COVID-19. Public Health. 2021.

18. Fares S, Elmnyer MM, Mohamed SS, Elsayed R. COVID-19 Vaccination Perception and Attitude among Healthcare Workers in Egypt. Journal of Primary Care & Community Health. 2021;12:21501327211013303.
19. Lin Y, Hu Z, Zhao Q, Alias H, Danaee M, Wong LP. Understanding COVID-19 vaccine demand and hesitancy: A nationwide online survey in China. PLoS neglected tropical diseases. 2020;14(12):e0008961.
20. Fisher KA, Bloomstone SJ, Walder J, Crawford S, Fouayzi H, Mazor KM. Attitudes toward a potential SARS-CoV-2 vaccine: a survey of US adults. Annals of internal medicine. 2020;173(12):964-73.
21. Dereje N, Tesfaye A, Tamene B, Alemshet D, Abe H, Tesfa N, et al. COVID-19 Vaccine hesitancy in Addis Ababa, Ethiopia: A mixed-methods study. medRxiv. 2021.
22. Griffin DS MG, Griffin DO. Adolescents trust physicians for vaccine information more than their parents or religious leaders. Heliyon. 2018;4(12):e01006.
23. Karafillakis E DI, Apfel F, et al. Vaccine hesitancy among healthcare workers in Europe: a qualitative study. Vaccines. 2016;34(41):5013- 20.
24. Reliefweb. Accelerating broad and equitable access to the COVID-19 vaccine in Ethiopia: https://reliefweb.int/report/ethiopia/accelerating-broad-and-equitable-access-covid-19-vaccine-ethiopia. 2021.
25. Kumar H, Khurana MS, Charan GS, Sharma NK. Knowledge and Perception of Health Professionals towards COVID-19. Age (years). 2020;25(090):11.5.
26. Verger P, Scronias D, Fradier Y, Meziani M, Ventelou B. Online study of health professionals about their vaccination attitudes and behavior in the COVID-19 era: addressing participation bias. Human Vaccines & Immunotherapeutics. 2021:1-6.
27. Islam MS, Siddique AB, Akter R, Tasnim R, Sujan MSH, Ward PR, et al. Knowledge, attitudes and perceptions towards COVID-19 vaccinations: a cross-sectional community survey in Bangladesh. medRxiv. 2021.
28. Verger P, Scronias D, Dauby N, Adedzi KA, Gobert C, Bergeat M, et al. Attitudes of healthcare workers towards COVID-19 vaccination: a survey in France and French-speaking parts of Belgium and Canada, 2020. Eurosurveillance. 2021;26(3):2002047.
29. Elhadi M, Alsoufi A, Alhadi A, Hmeida A, Alshareea E, Dokali M, et al. Knowledge, attitude, and acceptance of healthcare workers and the public regarding the COVID-19 vaccine: a cross-sectional study. BMC public health. 2021;21(1):1-21.
30. Ahmed NJ, Alkhawaja FZ, Alrawili AS, Almalki ZS. Public knowledge and attitudes toward COVID-19 Vaccination: A cross-sectional study. Medical Science. 2021;25(108):279-84.
31. Mesesle M. Awareness and Attitude Towards COVID-19 Vaccination and Associated Factors in Ethiopia: Cross-Sectional Study. Infection and Drug Resistance. 2021;14:2193-9.

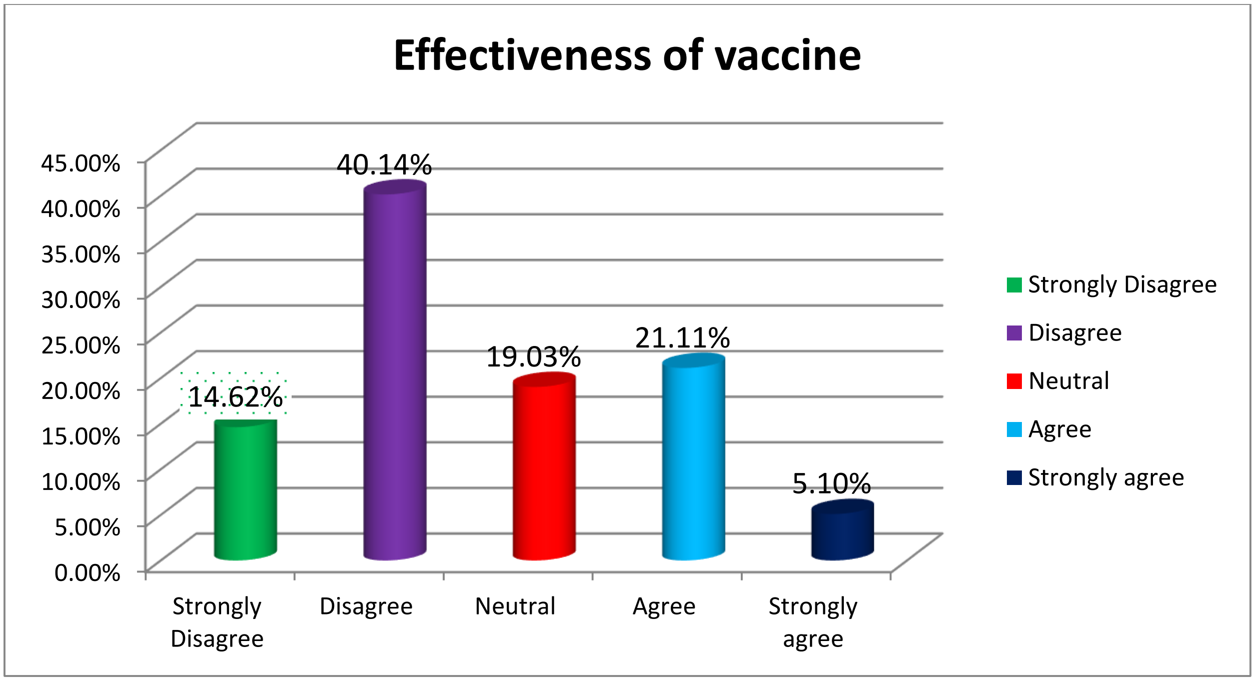

Figure 1: Attitude of the health professionals regarding effectiveness of the Corona virus vaccine

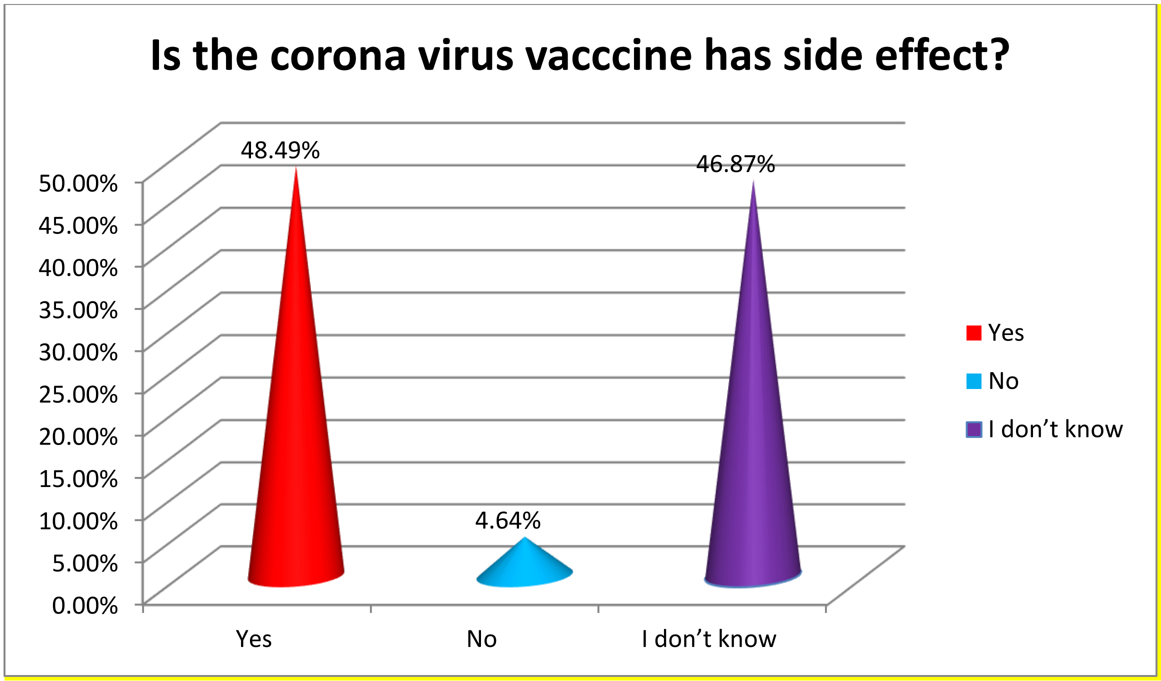

Figure 2: Perception the health care workers regarding Corona virus vaccine’s side effect
