# Supplementary material for: Attitude of health professionals towards COVID-19 vaccination and associated factors among health professionals, Western Ethiopia: A cross-sectional survey
Source: PLoS One. 2022 Mar 9;17(3):e0265061. doi: 10.1371/journal.pone.0265061 (PMC8906598; doi:10.1371/journal.pone.0265061)
Supplement: Attachment — Submitted filename: manuscript.docx [file pone.0265061.s004.docx]

**Attitude and perception of health professionals towards COVID-19 vaccination and associated factors among health professionals ~~found in Health facilities of Nekemte town,~~ Western Ethiopia**

**Tadesse Tolossa^1*^, Bizuneh Wakuma^2^, Ebisa Turi^1^, Diriba Mulisa^2^, Diriba Ayala^3^, Getahun Fetensa^2^, Belayneh Mengist^4^, Gebeyehu Abera^5^, Emiru Merdassa Atomssa^1^, Dejene Seyoum^1^, Tesfaye Shibiru^6^**

^*1^Department of Public Health, Institute of Health Science, Wollega University, Nekemte Ethiopia

^2^Department of Nursing, Institute of Health Science, Wollega University, Nekemte Ethiopia

^3^Department of Midwifery, Institute of Health Science, Wollega University, Nekemte, Ethiopia

^4^Department of Public Health, College of Health Science, Debre Markos University, Debre Markos, Ethiopia

^5^Nekemte Health Center, Nekemte, Ethiopia

^6^ School of Medicine, Institute of Health Sciences, Wollega University, Nekmete Ethiopia

**Corresponding author**: **Tadesse Tolossa^1*^**

*^1^***^*^** *Department of Public Health, Institutes of Health Sciences, Wollega University, Nekemte, Ethiopia*

E-mail- [yadanotolasa@gmail.com](mailto:yadanotolasa@gmail.com)

P.O.BOX: 395

Nekemte, Ethiopia

Tel: +251911936804

**Abstract**

**Introduction**: Even though people of the world were eagerly waiting for the hope of vaccine development, vaccine hesitancy is becoming the top concern in both developed and developing countries. However, there is no adequate evidence regarding the attitude and perception of health professionals towards the COVID 19 vaccine in resource-limited settings like Ethiopia. Hence, this study aimed to assess health professionals' attitudes and perceptions towards COVID 19 vaccine in West Ethiopia.

**Methods**: An institution-based cross-sectional study was conducted among health care workers found in Nekemte town from April 14-21, 2021. A total of 439 health professionals present on duty during the study period was included in the study. The data were collected by using a self-administered questionnaire.  Epidata version 3.2 was used for data entry, and STATA version 14 was used for data analysis. The binary logistic regression model was employed to determine factors associated with the attitude towards COVID-19 vaccination. Adjusted Odds Ratio (AOR) with 95% confidence intervals was computed, and statistical significance was declared at a 5% level (p-value < 0.05).

**Result**: A total of 431 health professionals were participated in the study yielding a response rate of 98.1%. The study indicates that 51.28% (95%CI: 45.12%, 57.34%) of health professionals had a favorable attitude towards COVID-19 vaccination. Having good knowledge about the COVID-19 vaccine (AOR=0.38, 95%CI: 0.22, 0.64) was negatively associated with unfavorable attitude towards COVID-19 vaccine, whereas working in a private clinic (AOR=7.77, 95% CI: 2.19, 27.58) and health center (AOR=2.45, 95%CI: 1.01, 5.92) were positively associated with the of unfavorable attitude towards COVID-19 vaccine.

**Conclusion and recommendation:** In general, the attitude and perception of health care professionals toward the COVID-19 vaccine in the study area were unsatisfactory. Poor knowledge about the COVID-19 vaccine and working in a private clinic and health center are the independent determinants of unfavorable attitudes towards the COVID-19 vaccine. Therefore, there is still a need to improve health professionals' knowledge of the COVID-19 vaccine by providing reliable information regarding vaccine safety, efficacy, and effectiveness.

**Keywords**: Attitude, COVID-19, Health professionals, vaccination, Ethiopia

# Introduction

Starting from the day it was declared a pandemic, and the COVID-19 remains the worst Global public health challenge. According to the worldometer report, the coronavirus COVID-19 affects about 220 countries and territories. More than 170 million cases and 3.5 million deaths happened due to COVID-19 (1, 2). The pandemic brought the double burden in developing countries already overwhelmed by the health care system challenges (3).

Given that sub-Saharan Africans are not the highest shareholder by cases and death during the early phase(4), the direct effect of COVID-19 and the indirect effect of its mitigation disrupted the health care services (5). The serious preventive measures (movement restriction, physical distancing, lockdowns, hand washing and sanitizing) were practiced (6). However, the counter effect of some mitigation brought significant change in health care settings, especially by task shifting and task sharing. Its effect on the economy is also evident. Though these mitigation procedures played a paramount role in averting the burden of COVID19, the whole world was hoping for vaccine development (7).

Many vaccines started to arise around 2020; there are hundreds of candidate vaccines (7). As of June 10, 2021, from 287 candidate vaccines, 102 are in the clinical phase, 185 are in the pre-clinical phase (8). From these, WHO has listed the Pfizer/BioNTech, Astrazeneca-SK Bio, Serum Institute of India, Janssen and Moderna vaccines for emergency use (6, 7). Believing HCWs are explicitly exposed to increased risk of infection through direct contact with patients, they should be prioritized for vaccination (9).

The willingness of the general population to accept the vaccine ahead of COVID-19 vaccine development was relatively promising compared to today's reality (10-12). Even though people of the world were eagerly waiting for the hope of vaccine development, vaccine hesitancy is becoming the top concern in both developed and developing countries (11, 13-17). For instance, the Vaccine acceptance is 21% in Egypt (18), 54.6% in China(19), and 57.6% of the adult population in the US(20) intended to be vaccinated. The most common reason mentioned for hesitancy were concerns about the safety of a vaccine and wide-ranging lack of confidence, worries about the efficiency of the vaccine(17, 18, 21)

Several evidence indicates that healthcare professionals (HCPs) play a paramount role and can significantly affect the general public's decisions to receive the COVID- 19 vaccine(22, 23). In Ethiopia, there have been 169,640 confirmed cases of COVID-19, with 9651 deaths till now, May 30, 2021 (1). The Ethiopian government and the Ethiopian Ministry of health exert great efforts to provide the COVID-19 vaccines and sort the vaccination as a priority for healthcare workers (HCWs) and older people, especially people with chronic diseases history (24). To overcome the expected upcoming challenge of vaccination hesitancy, we have to measure and know the exact reasons. However, there is no adequate evidence regarding the attitude and perception of health professionals towards the COVID-19 vaccine in resource-limited settings like Ethiopia. Hence, this study aimed to assess health professionals' attitudes and perceptions towards COVID 19 vaccine in West Ethiopia.

**Methods**

**Study area and period**

This study was conducted in health institutions found in Nekemte town. Nekmete is the capital city of East Wollega Zone, and it is located 330 KM from Addis Ababa, the capital city of Ethiopia. The study was conducted from April 15-21, 2021. Data from Nekemte Town Health Office showed that the town has one teaching referral hospital owned by Wollega University, one specialized hospital administered under Oromia Regional Health Bureau, two health centers (Nekemte and Cheleleki Health center), and more than fifteen medium and above private clinics. More than 800 health professionals are found in Nekemte town.

## Study design

An Institutional based cross-sectional study design was employed.

## Population, sample size and sampling techniques

All health professionals working in private and public health institutions of Nekemte town were a source population. Health professionals who were not on duty due to different reasons were excluded from the study. All health professionals on duty during data collection and willing to participate in the study were selected.

The sample size was determined by single population proportion with the following assumptions: Since this study was the first of its type in Ethiopia, p=50% was taken, with a 5% margin of error and a 95% confidence level. Accordingly, the calculated sample size was 384, and after adding a 15% allowance for a non-response rate, the final sample size was 439 health professionals.

All health institutions found in Nekemte town were included in the study regarding the sampling techniques. There are two public hospitals in Nekemte town, two health centers, and 15 medium clinics. Then the sample size was proportionally allocated to hospitals, health centers and private clinics. Around 710 health professionals are working in two hospitals, 50 in two health centers and 60 health professionals in all private clinics. For hospitals, we have used the identification number of the health professionals, and the identification number was used as a sampling frame. Then computer-generated simple random sampling technique was used to select the sample. Health professionals who were not on duty during the study period were excluded from the sampling frame. For health centers and private clinics, all health professionals who were available during the study period were included in the study. Furthermore, health professionals working in more than one health facility were considered only in a single health facility to prevent any distortion of information.

**Variables**

The attitude of health professionals towards the COVID-19 vaccine was the dependent variable of this study. Ten items were used to assess the attitude of health professionals towards COVID-19 cases. Each item was measured on a Likert scale with a five-point scale (strongly agree, agree, neutral, disagree, strongly disagree) to allow the study participants to express how much they agree or disagree with a particular question. "Favorable attitude" was when the scoring was ≥ mean score and less than mean value was rated as an "unfavorable attitude" towards COVID-19 vaccine. Socio-demographic variables such (age, sex, marital status, educational level, educational background, religion), medical history (chronic medical disease and previously infected with COVID-19), knowledge towards COVID-19 vaccine, and perception towards COVID-19 vaccine) were independent variables of this study. Five items assessed the knowledge of health professionals towards the COVID-19 vaccine. Each item contained the category of "Yes" and "No". A correct answer was assigned a "1" point, and an incorrect answer was assigned "0" points. The total score ranged from 0 to 5. "Good knowledge" was when the scoring was ≥ mean score and score below mean value indicated "poor knowledge" towards COVID-19 vaccine.

Finally, the perception of participants towards COVID-19 vaccine was assessed by using five items with "Yes"/No" category. Respondents who scored greater than or equal to the mean score were grouped to have "good perception" and participants who scored less than the mean score were grouped to have "poor perception" towards COVID-19 vaccine.

## Data collection techniques and data quality assurance

A questionnaire was developed by reviewing previously published papers (25, 26). The questionnaires comprised socio-demographic data, medical history, knowledge, attitude, and perception towards the COVID-19 vaccine. The data was collected by using self-administered questionnaires. The questionnaire was given to all health professionals on duty and returned to data collectors after filling it. The questionnaire was prepared in English, and the questionnaire's content was explained to them prior to distributing it.

The questionnaire was pre-tested on 5% of participants. Then possible amendments were done based on the findings. The discussion was held between investigators and data collectors, based on the pre-test result, and accordingly, some amendments were made. The data collectors gave the one-day training about the tool and data collection procedure. Data were checked daily for completeness, accuracy, clarity, and consistency by the supervisors and principal investigator. Any error or ambiguity, and incompleteness were corrected accordingly.

## Data management and analysis

Epidata version 3.0 was used for data entry and exported to STATA version 14.0 for further analysis. Descriptive statistics, like frequencies, percentages, mean and standard deviation were computed. Before analysis, data were cleaned and edited by using simple frequencies and cross-tabulation. Re-categorization of categorical variables and categorization of continuous variables was done. The assumption of the logistic regression model was checked before fitting it to the model. The binary logistic regression model was fitted to determine factors associated with COVID-19 vaccine acceptability. The multivariable logistic regression analysis included factors associated with the outcome variable at 20 % (p-value ≤0.20) significant level in the bivariable logistic regression analysis. Then crude and adjusted odds ratios and their corresponding 95% confidence intervals were presented in the final multivariable logistic regression table. AOR with 95% confidence intervals was computed, and statistical significance was declared when it was significant at a 5% level (p-value < 0.05). Correlation matrices checked multicollinearity (association between explanatory variables), and the model goodness of fit test was checked by Hosmer and Lemeshow test.

## Ethical considerations

The study was approved, and ethical clearance letters were obtained from Wollega University, Institute of Health Science Research Ethics review board. After approval, a permission letter was obtained from the administrative body of health facilities to respective clinics. Verbal consent was obtained from study participants, and the purpose of this study was stated to all participants. Everybody participated voluntarily in this study.

# Results

# Socio-demographic characteristics of the health professionals

Four hundred thirty-one health professionals were interviewed and yielded a response rate of 98.1%. More than half of the total participants, 268 (62.28%), were male. Regarding the age of the health professionals, nearly two-thirds of them, 274 (63.57 %), belong to less than 30 years old age group. More than half 253(58.07%) of the participants were protestant religion followers. Greater than three fourth of the health professionals who participated in this study were from hospital institutions 350 (81.21%) ([Table 1](c:\\pdfbuilder\\temp\\Result (3).docx))

**Table 1**: Socio demographic characteristics of the health professionals in Nekemte City, Western Ethiopia, 2021

| **Variables** | **Option** | **Frequency** | **Percent** |
| --- | --- | --- | --- |
| Age | <30years old | 274 | 63.57 |
|  | >=30years old | 157 | 36.43 |
| Sex | Male | 268 | 62.18 |
|  | Female | 163 | 37.82 |
| Marital status | Never married | 176 | 40.84 |
|  | Married | 243 | 56.38 |
|  | Separated | 8 | 1.86 |
|  | Others | 4 | 0.93 |
| Religion | Muslim | 73 | 16.94 |
|  | Protestant | 253 | 58.70 |
|  | Catholic | 10 | 2.32 |
|  | Orthodox | 71 | 16.47 |
|  | Others | 24 | 5.57 |
| Institution | Hospitals | 350 | 81.21 |
|  | Health center | 34 | 7.89 |
|  | Private | 47 | 10.90 |
| Education level | Diploma | 38 | 8.82 |
|  | First degree | 362 | 83.99 |
|  | Masters | 24 | 5.57 |
|  | Doctors | 2 | 0.46 |
|  | Specialty degree | 5 | 1.16 |
| Back ground | Nurses | 186 | 43.16 |
|  | Midwifery | 56 | 12.99 |
|  | Pharmacy | 32 | 7.42 |
|  | Medical practitioner | 47 | 10.90 |
|  | Public health | 41 | 9.51 |
|  | Anesthetists | 19 | 4.41 |
|  | MLS | 36 | 8.35 |
|  | Psychiatrics | 6 | 1.39 |
|  | others | 8 | 1.86 |

**Medical disorder related characteristics of the health professionals**

Thirty-three healthcare workers have a history of taking vaccination in their lives 33 (7.66%). Less than a quarter of the health care workers in this study have chronic diseases 19 (4.41%). Participants' most commonly reported chronic disease was hypertension 6 (46.15%). Again in this study, greater than half of the participants were vaccinated against coronavirus 240 (55.68%).

## The attitude of the health professionals regarding vaccination against COVID-19

In this study, almost half of the participants have a poor attitude toward COVID-19 vaccination 210 (48.72 %). One-third of the participants strongly agreed that COVID-19 could not be controlled without vaccination. Moreover, nearly one-third of participants agreed that mass vaccination against COVID-19 helps overcome the pandemic. Regarding the effectiveness of the newly discovered COVID-19 vaccination, less than a quarter of the participants strongly disagreed with 63 (14.62%)(**[Figure 1](c:\\pdfbuilder\\temp\\Result (3).docx))**.

**Table 2: Attitude of the health care workers regarding vaccination against COVID-19**

| **Variables** | **Option** | **Frequency** | **Percent** |
| --- | --- | --- | --- |
| The vaccine that is currently given in Ethiopia is the actual one that those innovative countries are taking | Strongly disagree | 114 | 26.45 |
|  | Disagree | 132 | 30.63 |
|  | Neutral | 81 | 18.79 |
|  | Agree | 79 | 18.33 |
|  | Strongly agree | 25 | 5.80 |
| If one person takes COVID-19 vaccination, it has a great contribution for other people | Strongly disagree | 65 | 15.08 |
|  | Disagree | 86 | 19.95 |
|  | Neutral | 53 | 12.30 |
|  | Agree | 155 | 35.96 |
|  | Strongly agree | 72 | 16.71 |
| I will take the vaccine if I get it without hesitation | Strongly disagree | 61 | 14.15 |
|  | Disagree | 183 | 42.46 |
|  | Neutral | 51 | 11.83 |
|  | Agree | 86 | 19.95 |
|  | Strongly agree | 50 | 11.60 |
| I encourage my family and others to take the vaccination | Strongly disagree | 58 | 13.46 |
|  | Disagree | 135 | 31.32 |
|  | Neutral | 61 | 14.15 |
|  | Agree | 127 | 29.47 |
|  | Strongly agree | 50 | 11.60 |
| COVID-19 cannot be controlled without vaccination | Strongly disagree | 85 | 19.72 |
|  | Disagree | 136 | 31.55 |
|  | Neutral | 65 | 15.08 |
|  | Agree | 111 | 25.75 |
|  | Strongly agree | 34 | 34 |
| COVID-19 vaccine is fairly distributed for all | Strongly disagree | 67 | 15.55 |
|  | Disagree | 122 | 28.31 |
|  | Neutral | 84 | 19.49 |
|  | Agree | 99 | 22.97 |
|  | Strongly agree | 59 | 13.69 |
| Mass vaccination can overcome the epidemic attach of the COVID-19 | Strongly disagree | 53 | 12.30 |
|  | Disagree | 90 | 20.88 |
|  | Neutral | 77 | 17.87 |
|  | Agree | 138 | 32.02 |
|  | Strongly agree | 73 | 16.94 |
| The best prevention method is to take the vaccine against COVID-19 | Strongly disagree | 54 | 12.53 |
|  | Disagree | 91 | 21.11 |
|  | Neutral | 92 | 21.35 |
|  | Agree | 134 | 31.09 |
|  | Strongly agree | 60 | 13.92 |
| The COVID-19 vaccine is not tested adequately for its effectiveness | Strongly disagree | 28 | 6.50 |
|  | Disagree | 73 | 16.94 |
|  | Neutral | 67 | 15.55 |
|  | Agree | 129 | 29.93 |
|  | Strongly agree | 134 | 31.09 |
| After being vaccinated against COVID-19 other precautions can be avoided | Strongly disagree | 89 | 20.65 |
|  | Disagree | 131 | 30.39 |
|  | Neutral | 62 | 14.39 |
|  | Agree | 66 | 15.31 |
|  | Strongly agree | 83 | 19.26 |
| The overall attitude towards a vaccine against COVID-19 | Favorable | 221 | 51.28 |
|  | Unfavorable | 210 | 48.72 |

**Perception the health care workers regarding COVID-19 vaccine**

| **Variables** | **Option** | **Frequency** | **Percent** |
| --- | --- | --- | --- |
| Have you ever been infected with COVID-19 | Yes | 95 | 22.04 |
|  | No | 169 | 39.21 |
|  | I don’t know | 167 | 38.75 |
| Do you think that the COVID-19 vaccine is effective? | Yes | 131 | 30.39 |
|  | No | 34 | 7.89 |
|  | I don’t know | 266 | 61.72 |
| Do you think that covid vaccine is mandatory for health care workers? | Yes | 262 | 60.79 |
|  | No | 262 | 60.79 |
|  | I don’t know | 145 | 33.64 |
| Do you think COVID-19 will be controlled only by preventive measures without vaccination? | Yes | 85 | 19.72 |
|  | No | 102 | 23.67 |
|  | I don’t know | 244 | 56.61 |
| Do you think that various COVID-19 vaccines have been discovered? | Yes | 142 | 32.95 |
|  | No | 46 | 10.67 |
|  | I don’t know | 243 | 56.38 |
| Do you think that the COVID-19 vaccine will be affordable and accessible by the common person! | Yes | 59 | 13.69 |
|  | No | 145 | 33.64 |
|  | I don’t know | 227 | 52.67 |

The distributions of each perception item about the COVID-19 vaccine are presented in Table 3. Concerning the question 'Do you think the COVID-19 vaccine is effective? Nearly one-third of them thought the vaccine against COVID-19 was effective. More than half of the participants responded unsure for the question 'Do you think COVID-19 will be controlled only by preventive measures without vaccination'? ([Table 3](c:\\pdfbuilder\\temp\\Result (3).docx)). Almost half of the participants accept that the newly discovered COVID-19 vaccine has side effect 209 (48.49%) ([Figure 2](c:\\pdfbuilder\\temp\\Result (3).docx))

**Table 3: Perception of the health care workers regarding COVID-19 vaccine, 2021**

# Factors associated with Attitude of health professionals toward COVID-19 vaccine.

In multivariable logistic regression, variables like age, sex, institution types, having chronic diseases and allergic reaction to previous medication have shown significant association with the outcome of interest. From those variables, two of them showed significant association with the attitude of health professionals toward a vaccine against COVID-19. The likelihood of having a poor attitude toward the COVID-19 vaccine was 2.45 times higher among Health professionals working at health centers than those working at hospitals AOR=2.45(95%CI 1.01,5.92). Similarly, the odds of having a poor attitude towards the COVID-19 vaccine was 7.77 times higher among health professionals working at private clinics and hospitals AOR=7.77(95% CI 2.19,27.58). Moreover, the proportion of health professionals with unfavorable attitudes was 62% lower among professionals who have good knowledge of COVID-19 vaccination than their counterparts AOR=0.38(95%CI: 0.22, 0.64) ([Table 4](c:\\pdfbuilder\\temp\\Result (3).docx)).

Table 4: **Multivariable analysis of factors associated with attitude towards COVID-19 vaccine among health professionals in Nekemte City, Western Ethiopia, 2021**

| **Characteristics** | | **Attitude** | | **COR(95%CI)** | **AOR(95%CI)** | **P-value** |
| --- | --- | --- | --- | --- | --- | --- |
|  |  | **Good** | **Poor** |  |  |  |
| Age | <30 | 125 | 149 | 1.87(1.25,2.79) | 2.14(1.25,3.67) | <0.01 |
|  | >=30 | 96 | 61 | Ref | | |
| Sex | Male | 143 | 125 | Ref | | |
|  | Female | 78 | 85 | 0.80 (0.54,1.18) | 0.93(0.54, 1.59) | 0.795 |
| Institution | Hospital | 155 | 195 | Ref | | |
|  | Health center | 24 | 10 | 3.01(1.40, 6.50) | 2.45(1.01,5.92) | 0.045* |
|  | Private clinic | 42 | 5 | 10.56 (4.0,27.35) | 7.77(2.19,27.58) | <0.01* |
| Chronic diseases | Yes | 10 | 9 | Ref | | |
|  | No | 211 | 201 | 0.94(0.37,2.37) | 0.76(0.25,2.29) | 0.630 |
| History of allergic reaction to previous medication | Yes | 20 | 21 | Ref | | |
|  | No | 201 | 189 | 1.11(0.58,2.12) | 1.04(0.46,2.33) | 0.914 |
| Knowledge about COVID-19 vaccination | Poor knowledge | 86 | 44 | Ref | | |
|  | Good knowledge | 163 | 139 | 0.30( 0.18,0.49) | 0.38(0.22,0.64) | <0.01* |

# Discussion

Though the vaccine development against COVID-19 was promising for the world population, vaccine hesitancy has become a global challenge to the successful uptake of the vaccine (11, 13-17). Works of literature are reporting as vaccine safety and efficacy are among the worries of the peoples and the reasons for vaccine hesitancy (17, 18, 21). Health care professionals are at a particular concern to be vaccinated to influence the general population for vaccination positively. There was a dearth of information about the attitude and perception of health professionals toward COVID-19 vaccination and its associated factor in Ethiopia, particularly in the study area. Therefore, this study was intended to determine the attitude and perception of health professionals toward COVID-19 vaccination and its associated factors in Nekemte town.

Accordingly, 31.55% of the study participants had agreed to take the vaccine without hesitation if available in the present study. This is lower than the study finding from Bangladesh (58.6%)(27), Canada (72.40)(28). The possible explanation for this might be the variation in trust and reliability of the source of information about the COVID-19 vaccine. Moreover, 41.07% of the study subjects in the present study have also agreed on encouraging families and others to take the vaccine, while 65.5% of participants in Bangladesh agreed to do so. Furthermore, 59.75% of this study's health professionals agreed that COVID-19 would not be controlled without vaccination. This echoes the finding from Bangladesh (63.4%)(27). In addition, nearly half of the study participants in the current study have agreed on mass vaccination to overcome the pandemic. This is also congruent with Elhadi M et al. in Libya(29). Comparable to the study finding from Saudi Arabia (37%)(30), only one-third of participants agreed on the vaccine's effectiveness in the present study. This depicts that there is still a need to provide reliable and accurate information about the vaccine's effectiveness against COVID-19 to health care professionals and the public.

The current study revealed that half of the health care professionals who participated in this study have a favorable attitude toward COVID-19 vaccination. This is lower than the study was done in Bangladesh reported that 78% of the general population had a favorable attitude(27). However, it is higher than the online survey was done in Ethiopia that reported 24.2% of the general population had a favorable attitude toward COVID-19 vaccination(31). The possible reason for the observed discrepancy might be due to the relative information difference among the population of interest. Furthermore, it might be due to health care professionals being at higher risk of infection with the virus that might result in a favorable attitude toward vaccination to minimize the risk of infection. This implies a need to assure the vaccine's effectiveness, safety, and efficacy to enhance its uptake by health professionals and the general public. Health professionals are the counselor and advocators of the general population that their decision to receive the vaccine can greatly influence the uptake by the general population.

The factors affecting health care professionals' attitude toward COVID-19 vaccination, knowledge toward COVID-19 vaccination, and type of health facility were independent determinants. Accordingly, the proportion of health professionals who have unfavorable attitudes was 62% lower among professionals with good knowledge of COVID-19 vaccination than their counterparts AOR=0.38(95%CI: 0.22, 0.64)). This might be because knowing the importance of the COVID-19 vaccine can positively influence someone to have a positive attitude toward it. Moreover, the odds of having unfavorable attitudes were much higher among health professionals working in private clinics and health centers than those in hospitals. This variation might be due to the relative number of client flow to the health center and private clinic being much lower than the hospital and, hence the professionals consider themselves at lower risk of exposure and infection to COVID-19. In addition, almost all COVID-19 patients have been admitted to hospitals than private clinics or health centers. Therefore, professionals working at health centers and private clinics for known and obvious reasons consider themselves to have a lower risk of infection with COVID-19. As a result, they might have an unfavorable attitude toward the COVID-19 vaccine than professionals working in the hospital setting.

**Limitation of the study**

The study did not provide qualitative perspectives on the attitude and perception of health professionals towards the COVID-19 vaccine and its associated factors, which, if available, could underpin the quantitative findings. Moreover, since it is a cross-sectional study, it did not address the cause and effect relationship between the factors and the outcome variables.

# Conclusion

In general, the attitude and perception of health care professionals toward the COVID-19 vaccine in the study area were unsatisfactory. Having poor knowledge about the COVID-19 vaccine, working in private clinics and health centers are the independent determinants of unfavorable attitudes towards the COVID-19 vaccine. Therefore, there is still a need to improve health professionals' knowledge of the COVID-19 vaccine by providing reliable information regarding vaccine safety, efficacy, and effectiveness. Furthermore, the media outlets need to work to develop trust among the public by disseminating accurate and consistent information about the vaccine. In addition, future researchers should also explore more about the attitude and perception of health care professionals toward the COVID-19 vaccine and its determinants using qualitative data.

# Abbreviations

**AOR**: Adjusted Odds Ratio: **COR**: Crude Odds Ratio, **HCW**: Health Care Worker, **MLS**: Medical Laboratory Science, **WURH**: Wollega University Referral Hospital

**Declaration**

## Ethical approval and consent to participate

The study was approved, and ethical clearance letters were obtained from Wollega University, Institute of Health Science Research Ethics review board. After approval, a permission letter was obtained from the administrative body of each health facility. Verbal consent was obtained from study participants, and the purpose of this study was stated to all participants. Everybody participated voluntarily in this study.

**Consent to publish**

Not applicable

**Availability of Data and Materials**

All data analyzed during this study are included in the manuscript.

**Competing Interests**

The authors declare that they have no competing interests.

**Funding**

No funding was obtained for this study.

**Authors' Contribution**

TT, BW, ET, GF, BM, DA, GA, and EM were involved in the initial development of the proposal. TT is involved in statistical analysis. TT, TS, and DS were involved in developing the initial drafts of the manuscript. All authors participated in the final preparation of the manuscript, and they approved the final draft of the manuscript for submission.

**Acknowledgments**

We want to thank all health facilities for their invaluable co-operation during data collection, and our deep acknowledgment also goes to the data collectors for their interest and commitment in carrying out the study.

**Author Information**

^*1^Department of Public Health, Institute of Health Science, Wollega University, Nekemte Ethiopia

^2^Department of Nursing, Institute of Health Science, Wollega University, Nekemte Ethiopia

^3^Department of Midwifery, Institute of Health Science, Wollega University, Nekemte, Ethiopia

^4^Department of Public Health, College of Health Science, Debre Markos University, Debre Markos, Ethiopia

^5^Nekemte Health Center, Nekemte, Ethiopia

^6^ School of Medicine, Institute of Health Sciences, Wollega University, Nekmete Ethiopia

# References

1.	Worldometer. COVID-19 Coronavirus Pandemic. Available online: https://[www.worldometers.info/coronavirus/?utmcampaign=homeAdvegas1?](http://www.worldometers.info/coronavirus/?utmcampaign=homeAdvegas1?) (accessed on May 30, 2021). 2021.

2.	Du R-H, Liang L-R, Yang C-Q, Wang W, Cao T-Z, Li M, et al. Predictors of mortality for patients with COVID-19 pneumonia caused by SARS-CoV-2: a prospective cohort study. European Respiratory Journal. 2020;55(5).

3.	Wachamo D, Tegene Y, Tibeso A, Washo A. Sexual and Reproductive Health Services Utilization and Associated Factors among College Students at West Arsi Zone in Oromia Region, Ethiopia. The Scientific World Journal. 2020;2020.

4.	Mehtar S, Preiser W, Lakhe NA, Bousso A, TamFum J-JM, Kallay O, et al. Limiting the spread of COVID-19 in Africa: one size mitigation strategies do not fit all countries. The Lancet Global Health. 2020;8(7):e881-e3.

5.	Muhaidat N, Fram K, Thekrallah F, Qatawneh A, Al-Btoush Aa. Pregnancy During COVID-19 Outbreak: The Impact of Lockdown in a Middle-Income Country on Antenatal Healthcare and Wellbeing. International Journal of Women's Health 2020;12:1065–73

6.	Mosisa G, Tsegaye R, Wakuma B, Mulisa D, Etefa W, Abadiga M, et al. Fertility desire and associated factors among people living with HIV in Ethiopia: a systematic review and meta-analysis. Archives of Public Health. 2020;78(1):1-16.

7.	Jasim SK. Post Cesarean Section Surgical Site Infection; Incidence and Risk Factors.

8.	World Health Organization COVID-19 vaccine tracker and landscape: https://[www.who.int/publications/m/item/draft-landscape-of-COVID-19-candidate-vaccines](http://www.who.int/publications/m/item/draft-landscape-of-covid-19-candidate-vaccines). 2021.

9.	Duch R, Roope LS, Violato M, Becerra MF, Robinson T, Bonnefon J-F, et al. Who should be first in line for the COVID-19 vaccine? Surveys in 13 countries of the publics preferences for prioritisation. medRxiv. 2021.

10.	Belsti Y, Gela YY, Akalu Y, Dagnew B, Getnet M, Seid MA, et al. Willingness of Ethiopian Population to Receive COVID-19 Vaccine. Journal of Multidisciplinary Healthcare. 2021;14:1233.

11.	Kaplan AK, Sahin MK, Parildar H, Guvenc IA. The willingness to accept the COVID‐19 vaccine and affecting factors among healthcare professionals: A cross‐sectional study in Turkey. International Journal of Clinical Practice. 2021:e14226.

12.	Dodd RH, Cvejic E, Bonner C, Pickles K, McCaffery KJ, Ayre J, et al. Willingness to vaccinate against COVID-19 in Australia. The Lancet Infectious Diseases. 2021;21(3):318-9.

13.	Alabdulla M, Reagu SM, Al‐Khal A, Elzain M, Jones RM. COVID‐19 vaccine hesitancy and attitudes in Qatar: A national cross‐sectional survey of a migrant‐majority population. Influenza and other respiratory viruses. 2021;15(3):361-70.

14.	Khubchandani J, Sharma S, Price JH, Wiblishauser MJ, Sharma M, Webb FJ. COVID-19 vaccination hesitancy in the United States: a rapid national assessment. Journal of Community Health. 2021;46(2):270-7.

15.	Lake EA, Demissie BW, Gebeyehu NA, Wassie AY, Gelaw KA, Azeze GA. Knowledge, attitude and practice towards COVID-19 among health professionals in Ethiopia: A systematic review and meta-analysis. PloS one. 2021;16(2):e0247204.

16.	Latkin CA, Dayton L, Yi G, Konstantopoulos A, Boodram B. Trust in a COVID-19 vaccine in the US: A social-ecological perspective. Social science & medicine (1982). 2021;270:113684.

17.	Troiano G, Nardi A. Vaccine hesitancy in the era of COVID-19. Public Health. 2021.

18.	Fares S, Elmnyer MM, Mohamed SS, Elsayed R. COVID-19 Vaccination Perception and Attitude among Healthcare Workers in Egypt. Journal of Primary Care & Community Health. 2021;12:21501327211013303.

19.	Lin Y, Hu Z, Zhao Q, Alias H, Danaee M, Wong LP. Understanding COVID-19 vaccine demand and hesitancy: A nationwide online survey in China. PLoS neglected tropical diseases. 2020;14(12):e0008961.

20.	Fisher KA, Bloomstone SJ, Walder J, Crawford S, Fouayzi H, Mazor KM. Attitudes toward a potential SARS-CoV-2 vaccine: a survey of US adults. Annals of internal medicine. 2020;173(12):964-73.

21.	Dereje N, Tesfaye A, Tamene B, Alemshet D, Abe H, Tesfa N, et al. COVID-19 Vaccine hesitancy in Addis Ababa, Ethiopia: A mixed-methods study. medRxiv. 2021.

22.	Griffin DS MG, Griffin DO. Adolescents trust physicians for vaccine information more than their parents or religious leaders. Heliyon. 2018;4(12):e01006.

23.	Karafillakis E DI, Apfel F, et al. Vaccine hesitancy among healthcare workers in Europe: a qualitative study. Vaccines. 2016;34(41):5013- 20.

24.	Reliefweb. Accelerating broad and equitable access to the COVID-19 vaccine in Ethiopia: https://reliefweb.int/report/ethiopia/accelerating-broad-and-equitable-access-COVID-19-vaccine-ethiopia. 2021.

25.	Kumar H, Khurana MS, Charan GS, Sharma NK. Knowledge and Perception of Health Professionals towards COVID-19. Age (years). 2020;25(090):11.5.

26.	Verger P, Scronias D, Fradier Y, Meziani M, Ventelou B. Online study of health professionals about their vaccination attitudes and behavior in the COVID-19 era: addressing participation bias. Human Vaccines & Immunotherapeutics. 2021:1-6.

27.	Islam MS, Siddique AB, Akter R, Tasnim R, Sujan MSH, Ward PR, et al. Knowledge, attitudes and perceptions towards COVID-19 vaccinations: a cross-sectional community survey in Bangladesh. medRxiv. 2021.

28.	Verger P, Scronias D, Dauby N, Adedzi KA, Gobert C, Bergeat M, et al. Attitudes of healthcare workers towards COVID-19 vaccination: a survey in France and French-speaking parts of Belgium and Canada, 2020. Eurosurveillance. 2021;26(3):2002047.

29.	Elhadi M, Alsoufi A, Alhadi A, Hmeida A, Alshareea E, Dokali M, et al. Knowledge, attitude, and acceptance of healthcare workers and the public regarding the COVID-19 vaccine: a cross-sectional study. BMC public health. 2021;21(1):1-21.

30.	Ahmed NJ, Alkhawaja FZ, Alrawili AS, Almalki ZS. Public knowledge and attitudes toward COVID-19 Vaccination: A cross-sectional study. Medical Science. 2021;25(108):279-84.

31.	Mesesle M. Awareness and Attitude Towards COVID-19 Vaccination and Associated Factors in Ethiopia: Cross-Sectional Study. Infection and Drug Resistance. 2021;14:2193-9.
