# Supplementary material for: Attitude of health professionals towards COVID-19 vaccination and associated factors among health professionals, Western Ethiopia: A cross-sectional survey
Source: PLoS One. 2022 Mar 9;17(3):e0265061. doi: 10.1371/journal.pone.0265061 (PMC8906598; doi:10.1371/journal.pone.0265061)
Supplement: Attachment — Submitted filename: Response to Reviewers.docx [file pone.0265061.s006.docx]

Dear Academic Editor of PLOS ONE journal

Dear Editor, this is regarding the manuscript PONE-D-21-21414 entitled as “Attitude and perception of health professionals towards COVID-19 vaccination and associated factors among health professionals found in Health facilities of Nekemte town, Western Ethiopia” submitted to PLOS ONE. Thanks for your time and consideration in editing and reviewing the manuscript. We have carefully read your comments and corrected inline of reviewer’s comments and suggestions. All comments raised were edited and incorporated in the main manuscript. Some of the changes were highlighted with yellow color in the manuscript. Here are the responses and elaborations for the comments!

Editor and Reviewer comments

The reviewers have raised a number of major concerns. They request improvements to the reporting of methodological aspects of the study, for example, providing more information on the tool used for data collection, the sample size calculation, and ensuring that the manuscript follows STROBE guidelines. In addition, the reviewers also note concerns about the quality of the written English and request that assistance is sought by a native English speaker with experience in scientific writing.

**Response**: Dear editor, thank you very much, we have addressed these all issues (tool, sample size, language) in the revised manuscript.

Journal Requirements:

When submitting your revision, we need you to address these additional requirements.

1. Please ensure that your manuscript meets PLOS ONE's style requirements, including those for file naming. The PLOS ONE style templates can be found at
   <https://journals.plos.org/plosone/s/file?id=wjVg/PLOSOne_formatting_sample_main_body.pdf> and 
   <https://journals.plos.org/plosone/s/file?id=ba62/PLOSOne_formatting_sample_title_authors_affiliations.pdf>

**Response:** Thank you Dear editor, we accepted your comment. All the revision was made in line with the journal requirements including the figure

2. Please include additional information regarding the survey or questionnaire used in the study and ensure that you have provided sufficient details that others could replicate the analyses. For instance, if you developed a questionnaire as part of this study and it is not under a copyright more restrictive than CC-BY, please include a copy, in both the original language and English, as Supporting Information.

**Response:** Thank you dear, we have addressed this issue in the method part of the manuscript

3. In your Data Availability statement, you have not specified where the minimal data set underlying the results described in your manuscript can be found. PLOS defines a study's minimal data set as the underlying data used to reach the conclusions drawn in the manuscript and any additional data required to replicate the reported study findings in their entirety. All PLOS journals require that the minimal data set be made fully available. For more information about our data policy, please see <http://journals.plos.org/plosone/s/data-availability>.

**Response:** Thank you, we have included minimal dataset in the revised manuscript as supplementary data

- please provide the reference number for the ethical approval clearance

**Response**: Thank you dear, we have included reference number in the revised manuscript
- please provide a completed STROBE checklist as an 'Other' file when resubmitting (see <https://www.strobe-statement.org/checklists/>)

**Response**: We have included as supplementary file

- please clarify why verbal consent was provided and not written informed consent.

**Response**: Dear editor, thank you for your question; however, written consent was not obtained from respondents. In Ethiopia, written consent is only possible if a sample is needed from the patients and any invasive procedure is performed. Since, we were not received any blood sample and invasive procedure were not performed; only verbal consent was obtained from participants.

**Reviewer #1:**

Dear Author,

I have some comments and corrections. I added an MS Word file.
You should edit your manuscript with STROBE guideline.
You should give exact values in the result section.
You should checked your manuscript for typos.

**Response**: Dear reviewer**,** Thank you for taking a time to review and edit our work thoroughly. Your effort in editing, and reviewing the overall document for its grammatical and vocabulary problem is very appreciable and valuable. Really, thank you in advance. We have tried to incorporate all your comments and corrections in the main manuscript.

**Reviewer #2:**

Generally, there are lots of grammar issues, incoherence of writing methods and results, as a result unreserved effort is recommended to the authors to increase the quality of the manuscript. All the comments are easily readable with Foxit reader in the manuscript.

**Response**: Dear Editor, we are very thankful for your important comment and we have tried to edit the grammatical flaws throughout the manuscript in its revised version. We have edited the spelling, grammatical errors, incomplete and poorly structured sentences throughout the manuscript. Now we believe the revised version is clean and clear enough to the readers.

1. There are lots of grammar issues in the manuscript, please correct it thoroughly?

**Response**: Thank you dear, we edited the grammatical error throughout the manuscript in its revised version

2. Two different results are reported in the abstract, which was associated with unfavorable attitude, was that good/poor knowledge ?

**Response**: Thank you dear, we have mentioned the the effect of knowledge on attitude towards the attitude in both result part and conclusion part, with the same message. In result part, it says good knowledge negatively associated with unfavorable attitude (implies having good knowledge about the vaccine could decrease the chance of poor attitude or good knowledge promote favorable attitude). In conclusion section it says poor knowledge positively affects unfavorable attitude, meaning lack of knowledge about the vaccine leads to poor attitude towards the vaccine. To make it clear, we have reported with the same statement in the revised manuscript.

3. One of your outcome, perception was not reported in the abstract section,why?

**Response**: Thank you dear, we have only one outcome variable (attitude). Perception was not the outcome variable, but we have used as an independent factor. To make it clear, we have removed perception from title in the revised manuscript.

4. Abbreviations/acronyms must be written in full form in first time writing?

**Response**: kindly accepted your comment

5. There is also a study in Ethiopian population which stating the willingness of Ethiopian population to take the vaccine , it is better to cite it here?

**Response**: Thank you dear, we have cited studies done in Ethiopia in the revised manuscript.

6. Rlease, correct the punctuation ?

**Response**: Thank you dear, we have corrected it

7. Repetition of the word ''working'', please delete one of them?

**Response**: Corrected

8. Your total population is 800. so, why don’t you take either 50% of the population or correction formula, even you can take all of the professionals?

**Response**: Dear reviewer, we are grateful for this important question. Yes the total population is 800, and the estimated sample size was 439. We used p=50% to estimate the sample size. Our fear to take 50% of the total population was there is scientific background which says use 50% if the total population is 800. From the thumb rule of estimating sample size, it is possible take 50% if the total population is 500. So rather taking 50%, it better to calculate by taking p=50%. In addition, we did not used correction formula, because correction is simply used to save resources. So if we used correction formula the sample size would be less than 250 which could decrease the power of the study.

So, to have a scientific justification and increase the power of the study we used single population proportion formula.

9. Even your expected to give/revisit those health workers who were not on duty during your data collection, why?

**Response**: Dear, thank you for the question. Duty the urgency of the finding for the research communities we were not revisited the HCW who were on duty leave during data collection. However, we acknowledged this issue in the limitation section.

10. Correct like '' the attitude of health professionals..''

**Response**: Thank you dear, we have corrected it.

11. Better to say attitude was takes as favorable when the over all score was greater than or equal to the≥ mean.

**Response**: corrected

12. Please add ''as'' after such.

**Response**: corrected

13. You should elaborate how do you develop your questions, adapted/adopted/validated/reliability?

**Response:** Thank you dear, we accepted your comment and incorporated in the revise manuscript

14. You should elaborate how do you develop your questions, adapted/adopted/validated/reliability?

**Response**: Thank you dear, we have accepted your comment and incorporated in the revised manuscript.

15. Was acceptability/willingness your objective or research question? factors must be fitted towards attitude/perception of COVID 19 vaccine?

**Response**: Dear reviewer, apologies, it is an technical error, and we have corrected it

16. How do you classify age like this? and use better symbols to make it more standard?

**Response:** Dear reviewer, thank you for the question. Of course, we need to categorize continuous data into categorical data based on the standards and references, but there was no standard to categorize age for health professionals. We simply categorized age into two categories by reviewing previous article.

17. It is not nearly less than quarter so your expression is not related with your report please correct it like ''only 19 or any other?

**Response**: Thank you dear, we have accepted your comment.

18. after saying half of, quarter of etc, it is better to add their respective number and percentage consequently?

**Response**: Thank you dear we have accepted your comment

19. Make it full table title ''where''? the table has grammar issues, correct them?

**Response:** Thank you dear, we have admitted your comment

20. grammar?

**Response**: corrected

21. Still you didn,t report the relationship b/n independent variables and perception towards COVID 19? or modify your title accordingly?

**Response**: Dear reviewer, thank you for this important comment, and we have removed perception from the title.

22. Since your interest variable unfavorable attitude it is better to start by unfavorable/poor attitude in your table?

**Response**: Thank you dear, we have accepted your comment and incorporated in the revised manuscript

23. You should report firstly to your research question/objective of your study, i.e ''attitude and perceptions of professionals towards	COVID 19''? This report is the willingness of the participants?

**Response**: thank you dear, we have accepted your comment. But, 31.5% is not magnitude of vaccine hesitancy, but the attitude of health professional towards the vaccine hesisatncy. It is one item which was used to assess the attitude of health professionals.

24. your reference system must be either Vancouver or Harvard?

**Response**: Kindly accepted your comment (Vancouver throughout the manuscript)

25. there is a study done in Ethiopia that showed the willingness to take the vaccine, so it is better to compare with it?

**Response**: Thank you dear we have reviewed and discussed our finding with previous studies conducted in Ethiopia

26. age < 30 years was significantly associated in your model, but you didn,t discuss it? in addition perceptions of the participants was not reported at all,so either modify your title or please adhere to your objective?

**Response:** Thank you dear, we have incorporated your concerns in the revised manuscript
