# Supplementary material for: Attitude of health professionals towards COVID-19 vaccination and associated factors among health professionals, Western Ethiopia: A cross-sectional survey
Source: PLoS One. 2022 Mar 9;17(3):e0265061. doi: 10.1371/journal.pone.0265061 (PMC8906598; doi:10.1371/journal.pone.0265061)
Supplement: Attachment — Submitted filename: response letter.docx [file pone.0265061.s007.docx]

Dear Academic Editor of PLOS ONE journal

Dear Editor, this is regarding the manuscript PONE-D-21-21414 entitled as “Attitude of health professionals towards COVID-19 vaccination and associated factors among health professionals found in Health facilities of Nekemte town, Western Ethiopia” submitted to PLOS ONE. Thanks for your time and consideration in editing and reviewing the manuscript. We have carefully read your comments and corrected inline of reviewer’s comments and suggestions. All comments raised were edited and incorporated in the main manuscript. Some of the changes were highlighted with yellow color in the manuscript. Here are the responses and elaborations for the comments!

**TITLE**

Please add the study design in the title, as per the STROBE Guidelines. You might like to revise the title as follows: ‘Attitude of health professionals towards COVID-19 vaccination and associated factors among health professionals in Western Ethiopia: a cross sectional survey’.

**Response**: revised

**ABSTRACT**

**Introduction**:

1. Please delete ‘the’ before towards in this sentence: “…..health professionals the towards the COVID 19 vaccine in resource-limited settings like Ethiopia.”

**Response**: corrected

2. Please change ‘Hence, this study aimed’ to ‘The aim of study was’ in the following sentence:  “Hence, this study aimed to assess health professionals' attitudes and perceptions towards COVID 19 vaccine in West Ethiopia.”

**Response**: corrected

3. The authors have written ‘Western Ethiopia’ in the title and ‘West Ethiopia’ in the abstract. Please keep the same terminology in the whole manuscript so revise the text thoroughly.

**Response**: corrected

Results:

4. Please delete ‘were’ in this sentence: “A total of 431 health professionals were participated…..”.

**Response**: corrected

5. Please change from: ‘The study indicates that…” to ‘The results indicated that..’

**Response**: corrected

6. Please report p values in the abstract.

**Response**: corrected

Conclusion:

7. The following statement is not included and supported by the results hence it could be removed:  “Therefore, there is still a need to improve health professionals' knowledge of the COVID-19 vaccine by providing reliable information regarding vaccine safety, efficacy, and effectiveness.”

**Response**: corrected

INTRODUCTION:

8. Please delete ‘and’ before ‘the’ in this sentence “…., and the COVID-19…’

**Response**: corrected

9. Please delete either ‘coronavirus’ or  ‘COVID-19’from this sentence: “According to the worldometer report, the coronavirus COVID-19…”.

**Response**: corrected

10. Please revise this sentence: “More than 170 million cases, and 3.5 million deaths happened due to COVID-19 [1, 2].” as “More than 170 million cases, and 3.5 million deaths have happened due to COVID-19, as of (add date/month/year) [1, 2].

**Response**: corrected

11. Please delete ‘health care system challenges;, which is given twice in this sentence: “The pandemic brought the double burden in developing countries already overwhelmed by the health care system challenges already overwhelmed by the health care system challenges [3].”

**Response**: corrected

12. The following information has become old so it could be omitted and you can name a few COVID vaccines that are being used globally or locally. “Many vaccines started to arise around 2020; there are hundreds of candidate vaccines [7]. As of June 10, 2021, from 287 candidate vaccines, 102 are in the clinical phase, 185 are in the preclinical phase [8]. From these, WHO has listed the Pfizer/BioNTech, Astrazeneca-SK Bio,Serum Institute of India, Janssen and Moderna vaccines for emergency use [6, 7].

**Response**: corrected

13. Please delete ‘hope of’ from this sentence: Even though people of the world were eagerly waiting for the hope of vaccine development…”

**Response**: corrected

14. Please delete ‘A’ before ‘studies’ in the following sentence: “A studies conducted in different regions of Ethiopia,….”

**Response**: corrected

15. Please revise “Several evidence indicates’ to ‘Several studies indicate’….

**Response**: corrected

16. Please delete ‘now’ in this sentence:  “…..9651 deaths till now, May 30, 2021 [1].”

**Response**: corrected

METHODS:

17. Please change ‘found’ to ‘located’ in this sentence: “This study was conducted in health institutions found in Nekemte town.”

**Response**: corrected

18. Please add a full stop / period (.) at the end of this sentence: More than 800 health professionals are found in Nekemte town

**Response**: corrected

19. Sample size: please double check the sample size with15% non-response, as my calculation is a little higher than 439 reported.

**Response**: Thank you dear, it is a editorial error and we have corrected it

20. Please report who was included/considered as ‘health professionals’. Was it every body working in hospitals or only doctors, nurses and AHPs.

**Response**: Thank you dear, health professionals were all health care workers in Nekemte town regardless of their profession.

21. Variable mean: The authors report that items were measured on 5 point Likert scale  and “Attitude was takes as “favorable” when the overall score was greater than or equal to the mean and less than mean value was rated as an "unfavorable attitude" towards COVID-19 vaccine.” Could you please what were scores for each point of the scale and what was the mean value of the scale scores.

**Response**: revised in the updated version of manuscript

22. Could you please either refer to relevant table or report 10 items used to measure the attitude and report whether these items were adapted from an earlier survey or developed at home. Please report sources if adapted.

**Response**: revised in the updated version of manuscript

23. Please either refer to relevant table or report 5 items used to assess the knowledge of health professionals towards the COVID-19 vaccine. Also, report whether these items were adapted from an earlier survey or developed at home. Please report sources if adapted.

**Response**: revised in the updated version of manuscript

24. Please either refer to relevant table or report 5 items used to measure the perception and report whether these items were adapted from an earlier survey or developed at home. Please report sources if adapted. Could you also report how yes or no were scored.

**Response**: revised in the updated version of manuscript

25. There is repetition of reporting language in which questionnaire was developed. “The tool was designed and distributed to respondents in English language…”.  AND “The questionnaire was prepared in English,..”. Please avoid repetition and revise the text.

**Response**: revised in the updated version of manuscript

26. Please report the acceptable level of Cronbach’s alpha in the following sentence: “Cronbach’s alpha was used to assess the reliability of the tool.”

**Response**: included in the revised version of manuscript

27. Please refer questionnaire included as supplementary material.

**Response**: Hyperlinked

Software:

28. Software: please report citations and references for Epidata and STATA software used.

**Response**: Cited

Ethics:

29. Please report the data and number of ethics approval by the Ethics Review Board.

**Response**: ethical clearance number is included in the revised manuscript

RESULTS

30. In the methods section it is reported that the questionnaire was given to health professionals for self- completion while the results section reports that the health professionals were interviewed. Please provide the correct information about how and who completed the questionnaires Either respondents or researchers or both.

**Response:** Thank you dear, we have corrected it

31. You might like to delete ‘Again in this study’ in this sentence: “Again in this study, greater than half of the participants were vaccinated against coronavirus 240 (55.68%).”

**Response**: corrected

32. You might like to remove ‘In this study’ from this sentence: “In this study, almost half 210 (48.72 %) of the participants have poor attitude toward COVID-19 vaccination.”

**Response**: corrected

33. Please change ‘discovered’ to ‘developed’ in the following sentence: “Regarding the effectiveness of the newly discovered COVID-19 vaccination,..”

**Response**: corrected

34. Table 3: please check the number of respondents for item 3 “Do you think that covid vaccine is mandatory for health care workers?” because the total number is higher than 341 (Yes 262, 60.79%, No 262, 60.79% and I don’t know 145, 33.64%.

**Response**: corrected

35. Please double check data (counts and %) in all tables.

**Response**: corrected

DISCUSSION

36. Please revise/change ‘Works of literature are reporting as vaccine..” to ‘Literature shows that as vaccine…

**Response**: corrected

37. Please change ‘the worries of the peoples’ to ‘the worries of the people…”

**Response**: corrected

38. Please avoid reporting results in the discussion section like ‘AOR=0.38(95%CI: 0.22, 0.64)).’

**Response**: corrected

REFERNCES:

39. Please report abbreviations of journal names as reported in the PubMed, if available.

**Response**: corrected
